# Paleo-polyploidization in Lycophytes

**DOI:** 10.1016/j.gpb.2020.10.002

**Published:** 2020-11-04

**Authors:** Jinpeng Wang, Jigao Yu, Pengchuan Sun, Chao Li, Xiaoming Song, Tianyu Lei, Yuxian Li, Jiaqing Yuan, Sangrong Sun, Hongling Ding, Xueqian Duan, Shaoqi Shen, Yanshuang Shen, Jing Li, Fanbo Meng, Yangqin Xie, Jianyu Wang, Yue Hou, Jin Zhang, Xianchun Zhang, Xiu-Qing Li, Andrew H. Paterson, Xiyin Wang

**Affiliations:** 1 Center for Computational Biology and Genomics, and School of Life Sciences, North China University of Science and Technology, Tangshan 063200, China; 2 National Key Laboratory for North China Crop Improvement and Regulation, Agriculture University of Hebei, Baoding 071001, China; 3 State Key Laboratory of Systematic and Evolutionary Botany, Institute of Botany, Chinese Academy of Science, Beijing 100093, China; 4 Fredericton Research and Development Centre, Agriculture and Agri-Food Canada, Fredericton, New Brunswick, E3B 4Z7, Canada; 5 Plant Genome Mapping Laboratory, University of Athens, Athens, GA 30602, USA; 6 Department of Plant Biology, University of Georgia, Athens, GA 30602, USA; 7 Department of Crop and Soil Science, University of Georgia, Athens, GA 30602, USA; 8 Department of Genetics, University of Georgia, Athens, GA 30602, USA

**Keywords:** Vascular plant, Lycophytes, Genome, Polyploidy, Evolution

## Abstract

**Lycophytes** and seed plants constitute the typical **vascular plants**. Lycophytes have been thought to have no paleo-polyploidization although the event is known to be critical for the fast expansion of seed plants. Here, genomic analyses including the homologous gene dot plot analysis detected multiple paleo-polyploidization events, with one occurring approximately 13–15 million years ago (MYA) and another about 125–142 MYA, during the **evolution** of the **genome** of *Selaginella moellendorffii*, a model lycophyte. In addition, comparative analysis of reconstructed ancestral genomes of lycophytes and angiosperms suggested that lycophytes were affected by more paleo-polyploidization events than seed plants. Results from the present genomic analyses indicate that paleo-polyploidization has contributed to the successful establishment of both lineages—lycophytes and seed plants—of vascular plants.

## Introduction

Extant vascular plants can be divided into two major types, the euphyllophytes (ferns and seed plants) and the lycophytes, which diverged as early as 410 million years ago (MYA) [1]. *Selaginella moellendorffii*, as a model lycophyte, has a dominant and complex sporophyte generation and vascular tissues with lignified cell types. The deciphered genome of *S. moellendorffii* is approximately 212.6 Mb, including two haplotypes of approximately 106 Mb [1]. Recently, the even smaller genome of *S. lepidophylla*, approximately 109 Mb, was deciphered [2].

Recursive polyploidizations or whole-genome duplications have been proposed as a key evolutionary driving force of seed plants, contributing to their divergence and rapid expansion [3], [4]. Different from seed plants, genomic analyses with various approaches including a discontiguous MegaBLAST did not find evidence of paleo-polyploidization in both *S. moellendorffii* and *S. lepidophylla* genomes [1], [5].

Seed plant genomes can be quite complex, at least partially due to recursive polyploidization and repetitive sequence accumulation. It is often difficult to perform a comprehensive analysis to understand their genome structure and evolution. In several instances, ancestral polyploidization was omitted from genome analysis, resulting in problematic interpretation of the structure, evolution, and/or functional innovation of whole genomes and key gene families [6], [7], [8], [9]. Recently, we developed a pipeline [10], including the generation of dot plot chart of homologous genes [11], to facilitate the analysis of complex genomes, especially those of plants. Analysis of the cucurbit genomes using this pipeline revealed an overlooked paleo-tetraploidization event, occurring ~100 MYA, in the common ancestor of Cucurbiticeae plants, which possibly contributed to the establishment and fast divergence of the whole family [10].

Here, we used our pipeline [10], including the homologous gene dot plot analysis [11], to analyze both *Selaginella* genomes and other related plant genomes [12]. We detected multiple polyploidization events in lycophytes.

## Results

### Gene colinearity

**Figure 1:**
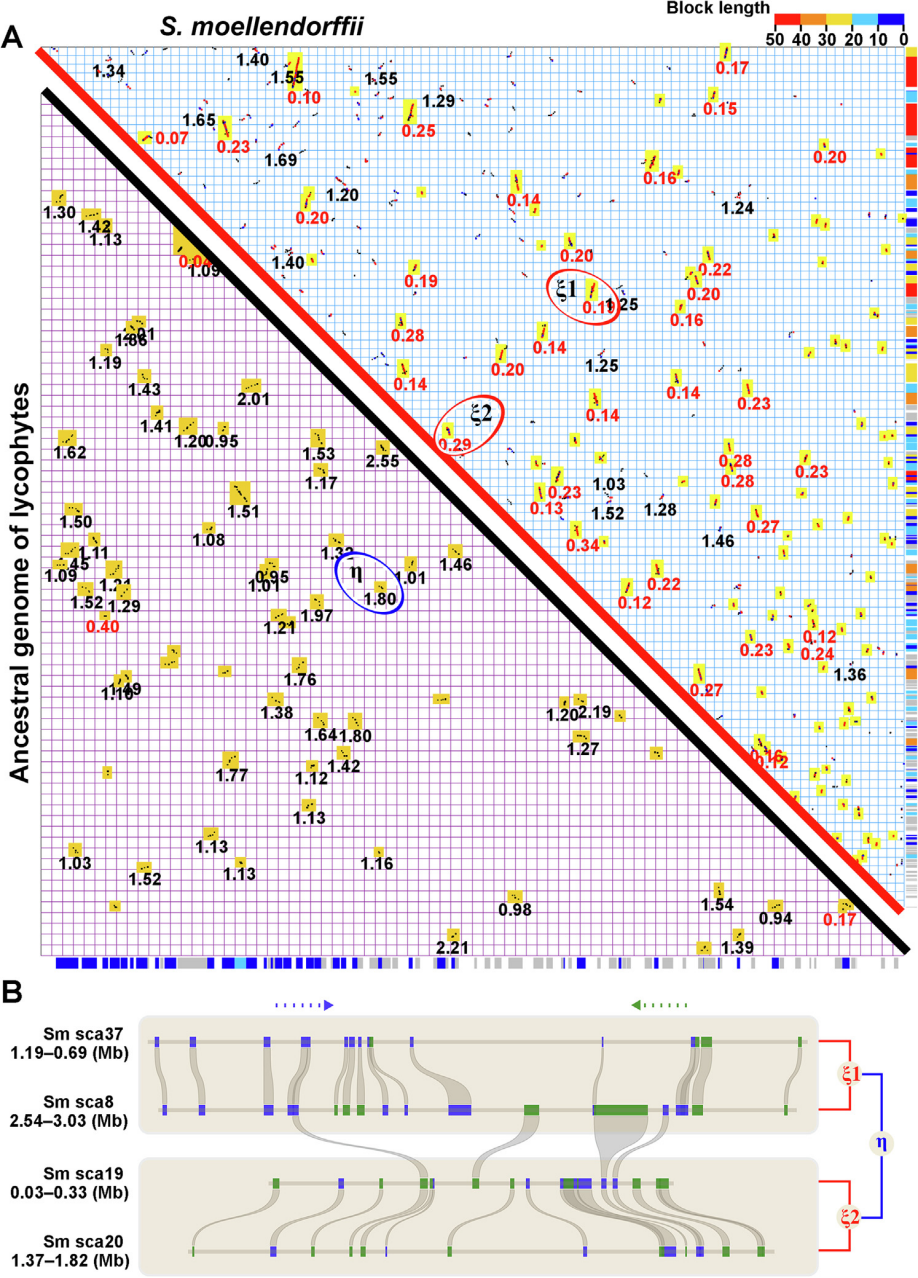
**Homologous gene dot plot of *Selaginella moellendorffii* and alignment of genomic regions with colinear genes** **A.** The upper-right diagonal shows dot plots between scaffolds of extant genomes. Both X- and Y-axes are represented by sequentially linked scaffolds. A duplicated gene pair inferred by ColinearScan between X and Y results in a dot. Neighboring dot plots result in blocks showing duplicated segments in the genome. Red, blue, and gray dots indicate best-, second-best, and other matches between genes. The median *K*s value of gene pairs in a block is displayed in red when *K*s < 0.5 and in black when 1 < *K*s < 1.7. Blocks involved in the recent tetraploidization, highlighted in yellow boxes, are mapped to the Y-axis, with the longest blocks in a neighboring region displayed with a color scheme and shown along the Y-axis to the right. The lower-left shows dot plots in the reconstructed ancestral genome before the recent tetraploidization. Dot plots are produced between genes in the ancestral genome. Blocks are highlighted, and *K*s values of blocks are displayed. The blocks are mapped to the X-axis, with the same color scheme defined above to show the longest blocks in a neighboring region. Examples of duplicated blocks produced by η and ξ are circled, to display gene colinearity in subfigure. **B.** The four regions involved are from *S. moellendorffii* scaffold20, scaffold19, scaffold8, and scaffold37, inferred to have been produced by two recursive polyploidizations, η and ξ. Genes are shown with rectangles, and the different colors show transcription directions. Colinear genes are linked with curved lines.

Using a gene-colinearity-based approach implemented in ColinearScan, with maximal gap between neighboring colinear genes of 50 genes (consistent with prior studies of many plants), we inferred 302 syntenic blocks in the *S. moellendorffii* genome (Tables S1 and S2). These blocks involved 2632 colinear gene pairs, and covered 87.01% (19,391/22,285) of all genes and 88.24% (19,391/21,975) of assembled genome sequences (Figure 1A). Use of a smaller gap size (25 genes) does not qualitatively change this result (65.92% genome coverage). The assembly had 192 scaffolds (of at least five genes), of which at least 75.52% (145/192) were covered by syntenic blocks with five or more colinear genes (more information about blocks can be found in Table S3).

The syntenic blocks in the *S. moellendorffii* genome were mapped onto the chromosomes and produced coverage as deep as 10. Approximately 19.24% of genome regions were covered with a depth of 4 or more, and 17.55%, 25.36%, 27.06%, and 10.79% of regions were covered with a depth of 3, 2, 1, and 0, respectively (Figure S1 and Table S4). Correspondence of colinear genes between multiple duplicated regions could often be found, showing probable recursive gene duplications (Figure 1B).

### Evidence of recursive polyploidization events

**Figure 2:**
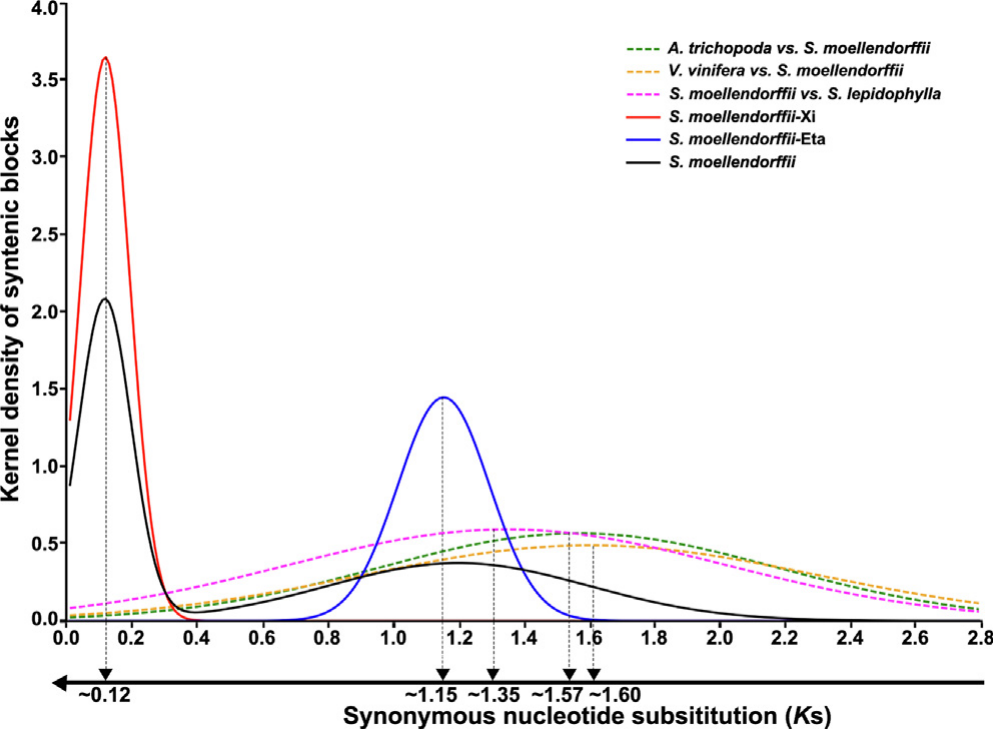
**Distribution of *K*s between colinear genes**

Sequence divergence of colinear genes in the *S. moellendorffii* genome clearly showed a non-random distribution consistent with the occurrence of polyploidization. Both synonymous nucleotide substitutions per synonymous site (*K*s) and nucleotide diversity for the fourfold synonymous third-codon transversion position (4Dtv) displayed clear bi-modal distributions (Figure 2, Figures S2 and S3). For *K*s, the two peaks were located at 0.12 and 1.2, respectively (Figure 2). Based on the *K*s distribution, we divided the colinear genes into two groups (Figure 1A). Supposing 6.0–7.0 × 10^−9^ synonymous substitutions per site per year, borrowed from angiosperms [3], [13], [14], the two peaks were re-posited at 0.12 and 1.15, respectively, suggesting large-scale genomic duplication events to have occurred ~13–15 MYA (named ξ or Xi) and ~125–142 MYA (named η or Eta). The use of separate distributions rather than the merged distribution allowed elimination of interactive effects during statistical analysis. The younger group covered 76.65% (16,844/21,975 genes) of the genome and 70.31% (135/192) of total scaffolds (related to all chromosomes). This result indicated that the event ξ was a polyploidization, with a *K*s peak clearly distinct from any residual heterozygosity of *S. moellendorffii* (DNA similarity ~98.5% [1], corresponding to *K*s ~0.056).

By merging the colinear gene blocks generated from the polyploidization event 13–15 MYA, we inferred that the ancestral genome before this event consisted of at least 120 syntenic blocks, which included 936 colinear genes, and covered 64.79% (8434/13,017) of the ancestral genome. We also discovered the existence of a more ancient whole-genome doubling event (the 125–142 η event), because self-comparison of these 120 blocks showed further colinearity that is extremely improbable to occur by chance.

### Comparison between ***S. lepidophylla*** and angiosperms

**Figure 3:**
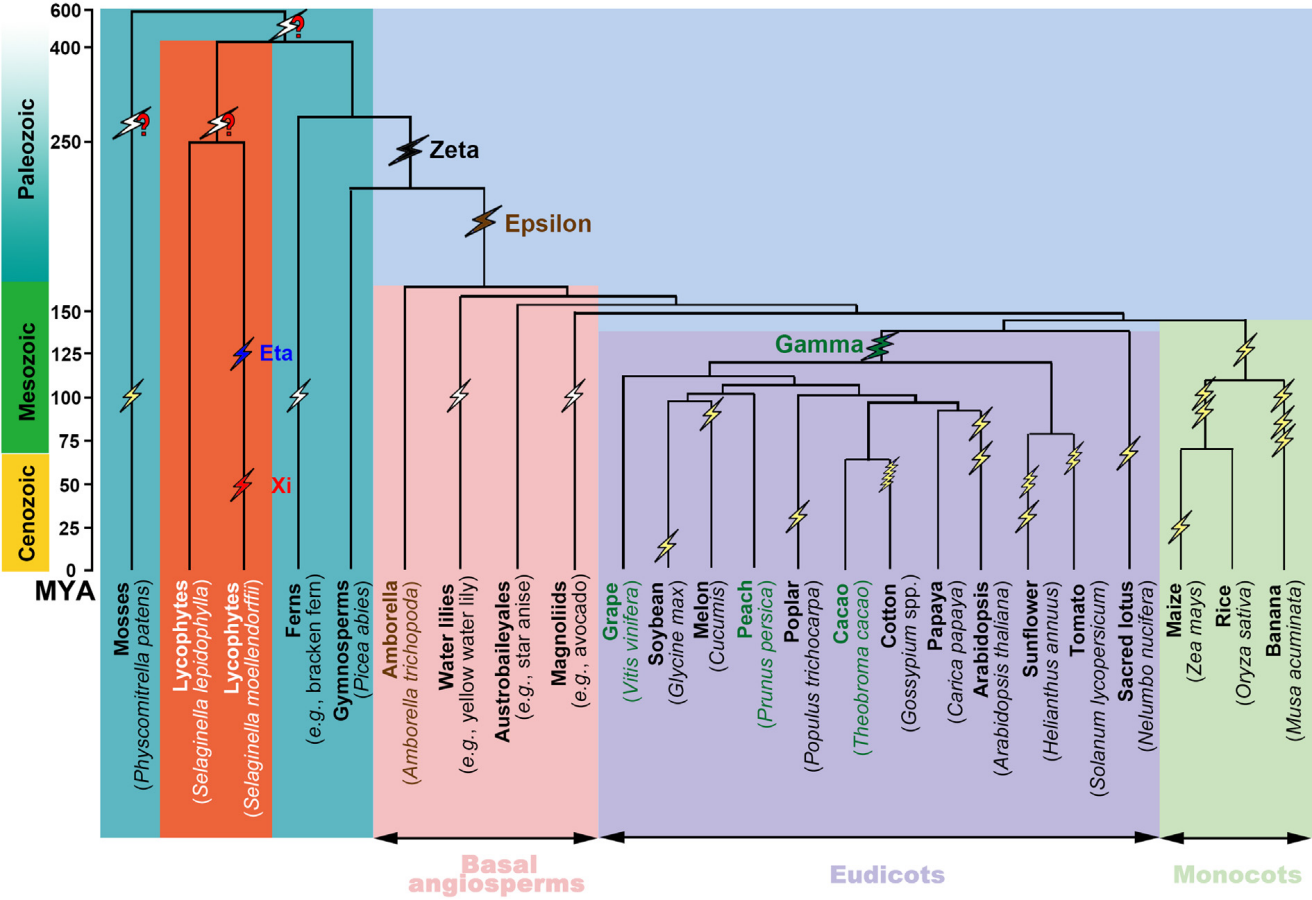
**Plant phylogeny and inferred ancient polyploidies** Flash marks are used to show polyploidizations, with two zigzags showing tetraploidy, three showing hexaploidy, five showing decaploidy, and a question mark indicating an undetermined ploidy level. The events η and ξ, inferred in the present work, are shown in blue and red, respectively. More ancient events that were inferred to have likely occurred are also shown with question marks.

Furthermore, we checked whether the two ancient polyploidization events detected in the *S. moellendorffii* genome was shared with *S. lepidophylla* and seed plants. Though two *Selaginella* genomes shared obvious gene colinearity (Tables S1 and S2), an analysis of the *S. lepidophylla* genome did not find evidence of polyploidization. The two lycophytes have 1121 colinear blocks with length ≥ 4 colinear genes, including 11,087 colinear genes in total. In *S. lepidophylla*, 110 blocks including 538 colinear genes were found, much fewer than in *S. moellendorffii*. By characterizing *K*s between colinear genes, we found *K*s peaks of putative orthologous genes at 1.35, 1.57, and 1.60 for *S. moellendorffii vs. S. lepidophylla*, *Amborella trichopoda* (a model angiosperm), and *Vitis vinifera* (grape), respectively (Figure 2). This result suggested that the two inferred polyploidization events in the *S. moellendorffii* lineage were absent in *S. lepidophylla* and in angiosperms (Figure 3).

### Existence of likely more paleo-polyploidization events

An appreciable fraction of *S. moellendorffii* genomic regions were covered at a depth > 4 by colinear blocks, suggesting more ancient polyploidization. Therefore, we performed a deeper search of gene synteny between *S. moellendorffii* and the two referenced angiosperm genomes. The *Amborella* genome, assembled into scaffolds, has a relatively simple structure and avoided a genome doubling after its split from other angiosperms, despite the existence of evidence of a shared ancient polyploidization [15] (Figure 3). The grape genome, well assembled into pseudochromosomes, was also used to reconstruct the ancestral genome before the major-eudicot-common hexaploidy. We inferred syntenic blocks between *S. moellendorffii* and each of the referenced genomes and mapped the blocks onto each of the genomes involved (Figures S4 and S5). Totals of 14.18% and 9.73% of the *S. moellendorffii* and *Amborella* genomes*,* respectively, were covered by colinear blocks to depth of 4 or more (Table S5). Likewise, 21.34% and 13.86% of the *S. moellendorffii* and grape genomes, respectively, were covered by colinear blocks to depth of 4 or more (Table S6). These findings suggested additional more ancient polyploidization events during the evolution of vascular plants.

**Figure 4:**
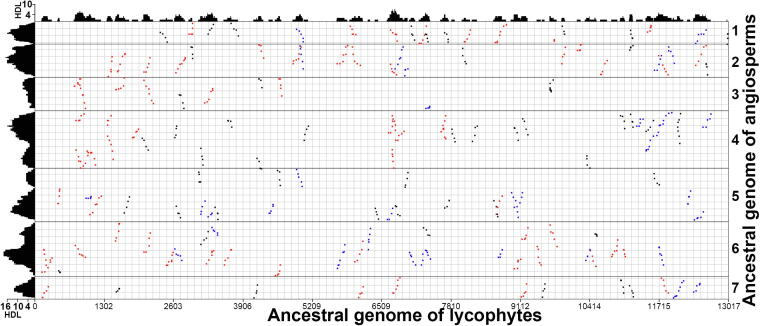
**Inferred colinear genes between ancestral genome of lycophytes (ALG) and ancestral genome of angiosperms (AAG)** Inferred ancestral regions represented by genes are arranged along X- and Y-axes, respectively. Statistically significant colinear blocks are displayed and mapped onto each axis to produce homologous coverage depth. Scale bars are displayed to the homologous depth level (HDL) in the genome. Blocks with median *K*s < 1.8 are colored red, blocks with *K*s ≥ 1.8 are colored blue, and others are colored gray.

We then explored the coverage depth of homologous regions in the reconstructed pre-ξ ancestral genome of lycophytes (ALG, 11,509 genes) and ancestral genome of angiosperms (AAG, 1686 genes) inferred on the basis of the grape genome. The ALG involved genes from the 34 largest *S. moellendorffii* scaffolds. At a gap size of 50 genes using ColinearScan, significant colinear blocks (*i.e.*, with at least four colinear genes) resulted in homologous coverage depth as deep as 7 and 18 in the AAG and ALG, respectively (Figure 4). Indeed, 78.28% and 7.49% of the AAG and ALG, respectively, were covered to depth of 4 or more (Table S7). To be careful, we tried different maximal gap sizes in each compared lineage (Table S7) and obtained similar conclusions (Figure 3).

In that *S. lepidophylla* was not affected by polyploidization after its divergence with *S. moellendorffii*, it could provide insight into polyploidization(s) older than their split from angiosperms. Therefore, we managed to use *S. lepidophylla* genomic DNA, though not as well assembled as that of *S. moellendorffii,* to infer gene colinearity. The *S. lepidophylla* contigs were sorted as to their best matched *S. moellendorffii* scaffolds. We found appreciable amount of colinearity within the genome itself, and between *S. lepidophylla* and AAG (Figure S6 and Table S1). Regarding coverage depth, 68.55% and 20.74% of the AAG and *S. lepidophylla*, respectively, were covered to depth of 4 or more (Table S8). At least 40% of the inferred colinear blocks terminate at the ends of assembled scaffolds, presumably broken by incomplete assembly rather than by lack of colinearity (Figure S7).

Since lycophytes and seed plants are thought to have diverged 410 MYA [1], and the two *Selaginella* plants (*S. moellendorffii* and *S. lepidophylla*) diverged approximately 250 MYA [2], the present findings of at least two paleo-polyploidization events in less than 150 MYA suggest that there may have been additional paleo-polyploidizations in each lineage of lycophytes and seed plants, separately. Moreover, greater coverage mapped onto the AAG means that lycophytes might have experienced more polyploidizations than seed plants.

We have to note that a more precise determination of the numbers and dates of these ancient polyploidization events is beyond the resolution of *K*s. Other sequence divergence analysis approaches using likely more taxa of basal vascular plants will be required.

## Discussion

Polyploidization or whole-genome duplication was inferred to have contributed to the early divergence of seed plants according to phylogenetic tree reconstruction [4]. By dissecting features of *K*s distribution with transcriptome data, horsetail, a basal land plant, was found to be an ancient polyploid [16]. A large-scale cytogenetic and phylogenetic analysis indicated that ferns might have been more frequently affected by polyploidization [17]. Together with many other publications proposing ancient polyploidization [18], [19], [20], [21], these works each shed light on the distant past of plant evolution. However, although the first *Selaginella* genome has been available for years, genome-scale evidence of ancient whole-genome duplication in the lycophytes remains elusive. Here, using the sophisticated pipeline that we developed for deciphering complex genomes, we provided clear and solid evidence that two polyploidization events have contributed to the evolution of the lycophyte *S. moellendorffii* after its split from *S. lepidophylla*, and several older polyploidization events likely shared also by *Selaginella* plants have occurred in the early days of vascular plants.

Polyploidizations were proposed to be the likely answer to the mystery about the abrupt origin and fast divergence of seed and flowering plants on Earth raised by Charles Darwin [20]. The present finding that lycophytes were also recursively affected by polyploidizations may benefit further evaluation how these events contributed to the expansion of different lineages of vascular plants.

The availability of whole-genome sequences provides evidence of ancient polyploidy. However, it is technically challenging to deconvolute these ancient large-scale events [6], [7], [8], [9]. The MegaBLAST analysis of the *Selaginella* genome overlooked these large-scale evolutionary events [1], possibly due to the complicated nature of plant genomes and/or drawbacks in the methodology adopted [10]. To decipher the complex genomes, genome dot plot often makes a good illustration of gene synteny/colinearity. Divergence of inferred colinear genes reveals non-random patterns that distinguish subsets with different ages. The exploration of colinear genes can provide insight into more ancient events, as proposed, and can be successfully used to decipher recursive polyploidizations during the evolution of eudicots based on the small genome of *Arabidopsis* [11]. Methodology that lacks inference of gene colinearity or use of homologous gene dot plots, *e.g.*, based largely on inferences of *K*s distribution or phylogenetic tree topology, could easily overlook evidence of paleo-polyploidization. Even *K*s distributions that give the appearance of polyploidization could be caused by genes duplicated by other events, such as waves of retrotransposition. The combination of temporal (*K*s) and positional (colinearity) evidence can provide for falsification of alternative hypotheses, to uncover ancient, especially recursive polyploidizations in extant complex genomes. Future research may help to understand how these polyploidization events have contributed to biological and genetic innovations during evolution.

## Conclusion

A major lineage of land plants, lycophytes, was thought to have avoided paleo-polyploidization. Here, using a sophisticated comparative genomics approach, we inferred that the genome of the first sequenced lycophyte *S. moellendorffii* was affected by at least two rounds of paleo-polyploidization events; and more analyses with another lycophyte and seed plants suggested more large-scale genome duplications during the evolution of land plants.

## Materials and methods

### Materials

Genome sequences and annotations were downloaded from the Joint Genome Institute (JGI, https://phytozome.jgi.doe.gov/pz/portal.html; *S. moellendorffii*, *S. lepidophylla*, *A. trichopoda*, and *V. vinifera L.*).

### Gene colinearity

The pipeline to decipher complex genomes was followed as described previously [10]. Homologous gene dot plots within a genome or between genomes were produced by using MCSCAN toolkits [22], of which the corresponding author directly contributed to the development. Colinear genes were inferred by using ColinearScan [23], a statistically well-supported software in pairwise homology. ColinearScan implements a modified dynamic programming algorithm and recursively searches the remaining longest path with colinear gene pairs as nodes. The maximal gap between genes involved in colinearity along a chromosome was evaluated computationally, and each colinear block inferred was then statistically assessed of their significance. Here, the maximal gap was set as 50 genes apart, and repetitive genes with > 30 copies were removed from the present analysis, as adopted in many previous studies [6], [24], [25], [26], [27]. Evolutionary divergence between homologous genes was estimated as described previously [24], [26].

### Analysis of *K*s

The synonymous nucleotide substitutions (*K*s) between duplicated genes in colinearity resulted from each of considered polyploidization and divergence events were analyzed. *K*s values were estimated using Nei-Gojobori approach by implementing the program codeml in the “phylogenetic analysis by maximum likelihood” software PAML [28], [29]. Gene CDS alignment was performed using ClustalW with default parameters [30].

### Construction of ancestral genomes

Construction of ancestral genomes was also based on our previously detailed methods [10], [11]. For clarification, here, we reconstructed the ancestral genome of lycophytes (ALG) before the polyploidization ξ, and the ancestral genome of angiosperms (AAG). The ALG was reconstructed by utilization of one copy of the colinear genes produced by the recent polyploidy, in that the colinear genes probably preserved their ancestral gene locations (Figure S8A). In a similar approach, the AAG genome was reconstructed by inferring ancestral genes using grape colinear genes produced by the major-eudicot-common hexaploidy (previously named ϒ) (Figure S8B)**.** We did not infer AAG using a grape-*Amborella* or grape-rice comparison due to incomplete assembly of the *Amborella* genome and severe genomic fractionation after multiple polyploidies in monocot lineages [31].

## CRediT author statement

**Jinpeng Wang:** Writing - original draft, Supervision, Methodology, Software. **Jigao Yu:** Software, Formal analysis, Data curation, Visualization. **Pengchuan Sun:** Software, Formal analysis, Visualization. **Chao Li:** Software, Formal analysis. **Xiaoming Song:** Software, Formal analysis, Data curation. **Tianyu Lei:** Software, Formal analysis. **Yuxian Li:** Software, Formal analysis, Data curation. **Jiaqing Yuan:** Software, Formal analysis, Data curation. **Sangrong Sun:** Formal analysis, Data curation. **Hongling Ding:** Formal analysis, Data curation. **Xueqian Duan:** Formal analysis, Data curation. **Shaoqi Shen:** Formal analysis, Data curation. **Yanshuang Shen:** Formal analysis, Data curation. **Jing Li:** Formal analysis, Data curation. **Fanbo Meng:** Investigation, Data curation. **Yangqin Xie:** Investigation, Data curation. **Jianyu Wang:** Investigation, Data curation. **Yue Hou:** Data curation. **Jin Zhang:** Data curation. **Xianchun Zhang:** Validation. **Xiu-Qing Li:** Writing - review & editing. **Andrew H. Paterson:** Writing - review & editing. **Xiyin Wang:** Conceptualization, Writing - original draft, Writing - review & editing, Supervision. All authors read and approved the final manuscript.

## Competing interests

The authors have declared no competing interests.

## Supplementary material

Supplementary Figure S1 **Homologous gene dot plot within *S. moellendorffii* genome**. All scaffolds were arranged along X- and Y-axes, respectively. Statistically significant collinear blocks are displayed, and they are mapped onto both axes to produce homologous coverage depth. Scale bars are displayed to the homologous depth level (HDL) in the genome.

Supplementary Figure S2 **Distribution of Ks between colinear genes.**

Supplementary Figure S3 **Distribution of four-fold degenerative transversions on the third codon sites between homologous genes within a genome or between genomes.**

Supplementary Figure S4 **Homologous gene dot plot between *S. moellendorffii* and *A. trichopoda***. Their scaffolds were arranged along Y- and X-axes, respectively. Statistically significant collinear blocks are displayed, and they are mapped onto both axe to produce homologous coverage depth. Scale bars are displayed to the homologous depth level (HDL) in the genome.

Supplementary Figure S5 **Homologous gene dot plot between *S. moellendorffii* and *V. vinifera***. Their scaffolds were arranged along X- and Y-axes, respectively. Statistically significant collinear blocks are displayed, and they are mapped onto both axe to produce homologous coverage depth. Scale bars are displayed to the homologous depth level (HDL) in the genome.

Supplementary Figure S6 **Homologous gene dot plot between *S. lepidophylla* and ancestral angiosperm genomes, *i.e.*, AAG**. *S. lepidophylla* genes and inferred ancestral angiosperm regions represented by genes are arranged along X- and Y-axes, respectively. Statistically significant colinear blocks are displayed and mapped onto each axis to produce homologous coverage depth. Scale bars are displayed to the homologous depth level (HDL) in the genome. Blocks with median *K*s < 1.8 are colored red, blocks with *K*s ≥ 1.8 colored blue, and others colored gray.

Supplementary Figure S7 **Homologous gene dot plot of *S. moellendorffii***. Blocks that come to the ends of assembled scaffolds are highlighted in blue, details in Figure S1.

Supplementary Figure S8 **Inference of ancestral genomes in Lycophytes and angiosperm lineages**. **A.** The pre-ξ ancestral genome of lycophytes is inferred by merging collinear *S. moellendorffii* genes produced by ξ. **B.** The ancestral genome of angiosperms is inferred by merging collinear grape genes produced by the major-eudicot-common hexaploidy.

Supplementary Table S1 **Number of colinear blocks and gene pairs within a genome or between genomes.**

Supplementary Table S2 **Gene number of syntenic blocks within genome or between genomes.**

Supplementary Table S3 **Block information.**

Supplementary Table S4 **Homology depth in the *S. moellendorffii* genome.**

Supplementary Table S5 **Homology depth between *S. moellendorffii* and *A. trichopoda* genomes.**

Supplementary Table S6 **Homology depth between *S. moellendorffii* and *V. vinifera* genomes.**

Supplementary Table S7 **Homology depth between ALG and AAG genomes.**

Supplementary Table S8 **Homology depth between *S. lepidophylla* and AAG genomes.**
