# Supplementary figures and images for: Paleo-polyploidization in Lycophytes
Source: Genomics Proteomics Bioinformatics. 2020 Nov 4;18(3):333–40. doi: 10.1016/j.gpb.2020.10.002 (PMC7801247; doi:10.1016/j.gpb.2020.10.002)

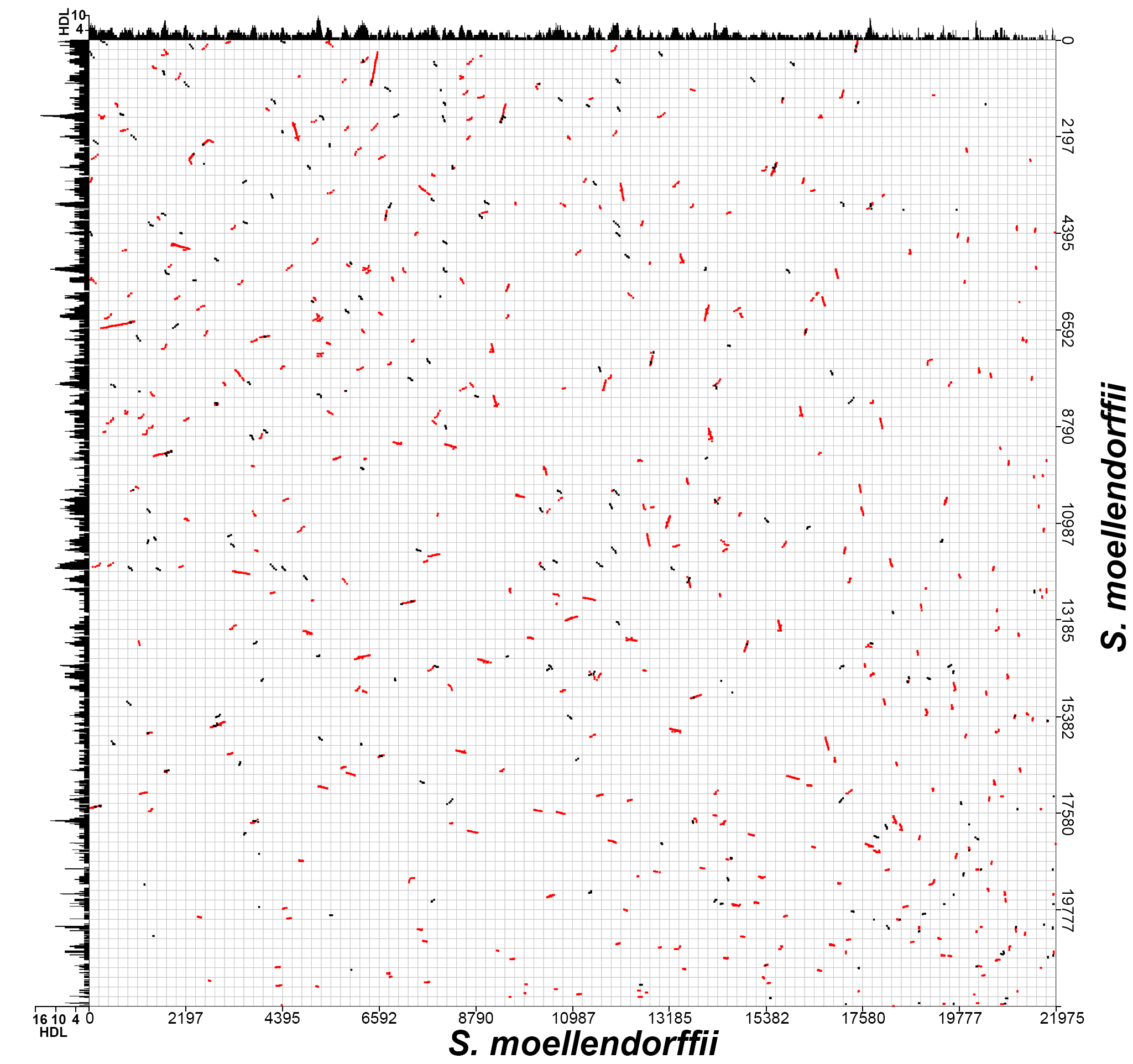

Supplement: Supplementary Figure S1 — Homologous gene dot plot within S. moellendorffii genome. All scaffolds were arranged along X- and Y-axes, respectively. Statistically significant collinear blocks are displayed, and they are mapped onto both axes to produce homologous coverage depth. Scale bars are displayed to the homologous depth level (HDL) in the genome. [file mmc1.zip › Figure S1 Au060120.png]

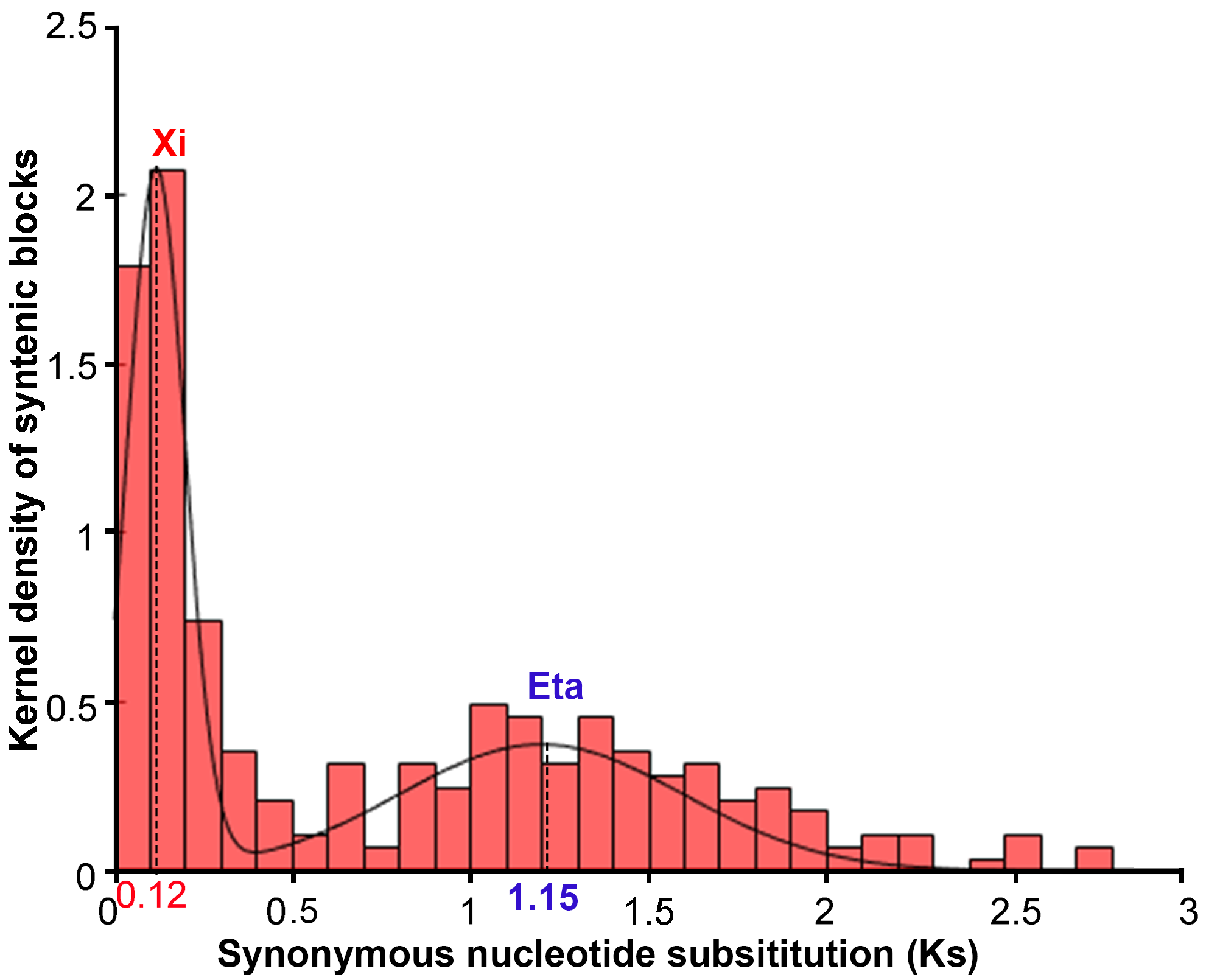

Supplement: Supplementary Figure S2 — Distribution of Ks between colinear genes. [file mmc2.zip › Figure S2 Au060120.png]

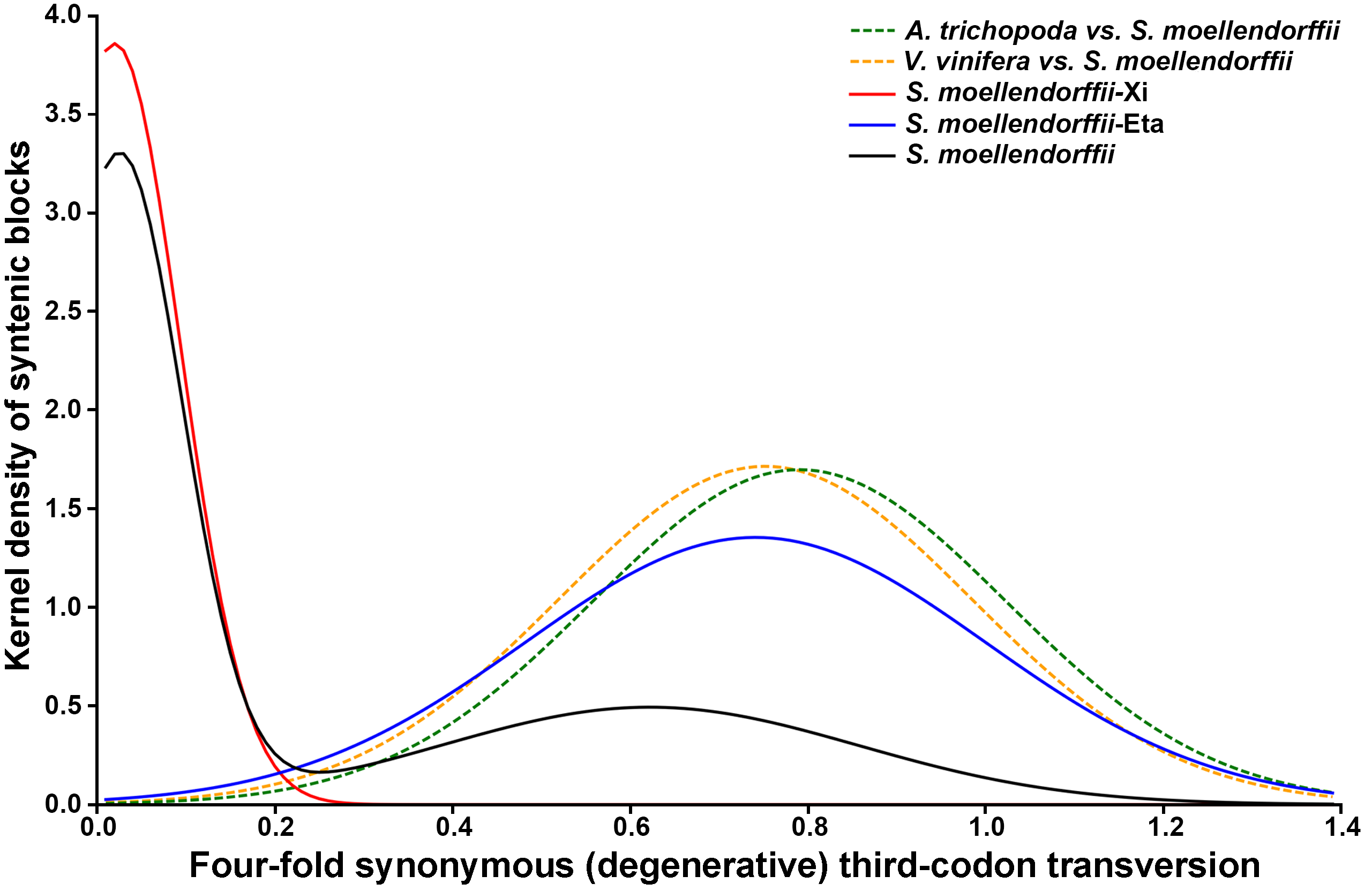

Supplement: Supplementary Figure S3 — Distribution of four-fold degenerative transversions on the third codon sites between homologous genes within a genome or between genomes. [file mmc3.zip › FIgure S3 Au060120.png]

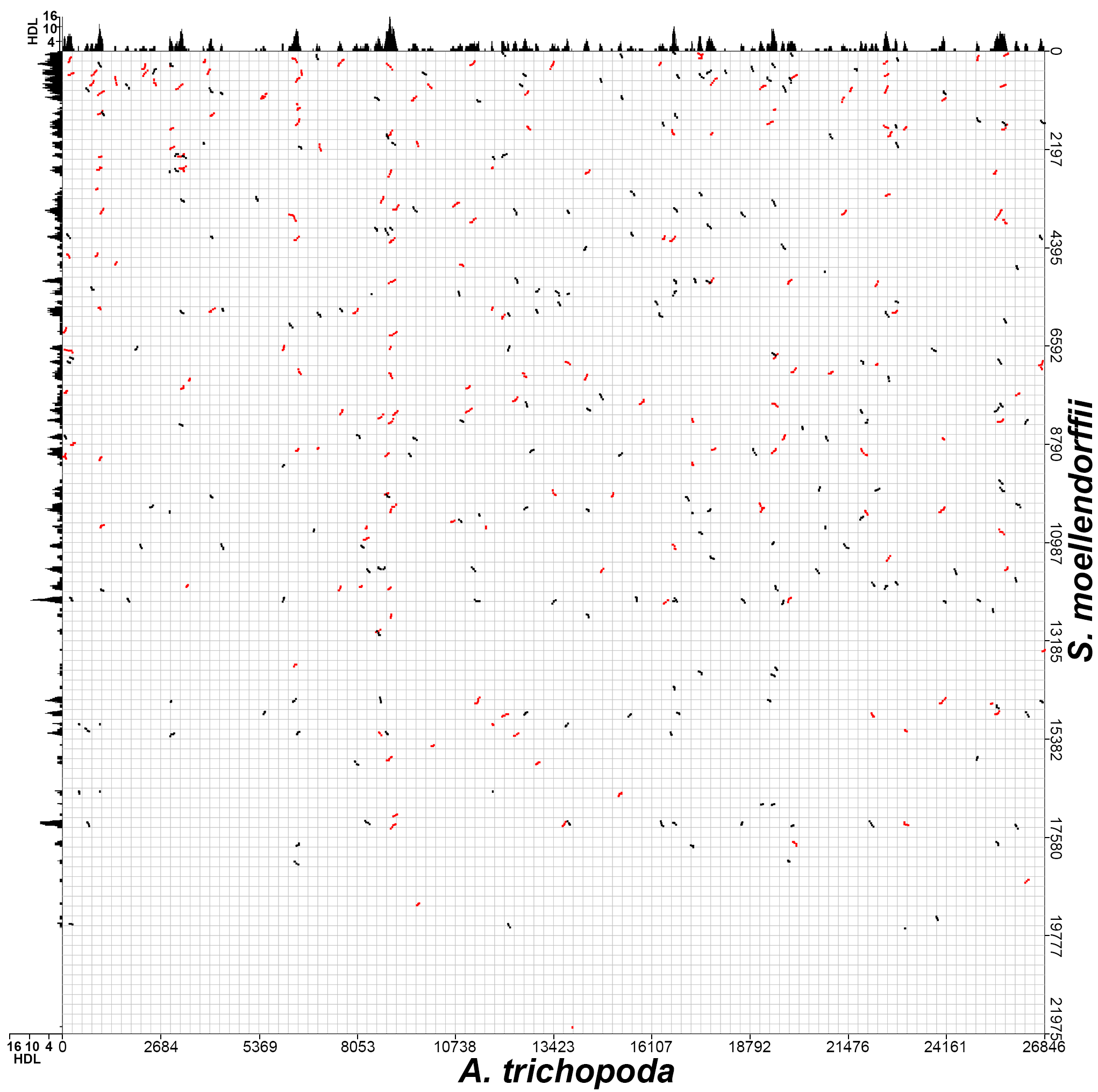

Supplement: Supplementary Figure S4 — Homologous gene dot plot between S. moellendorffii and A. trichopoda. Their scaffolds were arranged along Y- and X-axes, respectively. Statistically significant collinear blocks are displayed, and they are mapped onto both axe to produce homologous coverage depth. Scale bars are displayed to the homologous depth level (HDL) in the genome. [file mmc4.zip › Figure S4 Au060120.png]

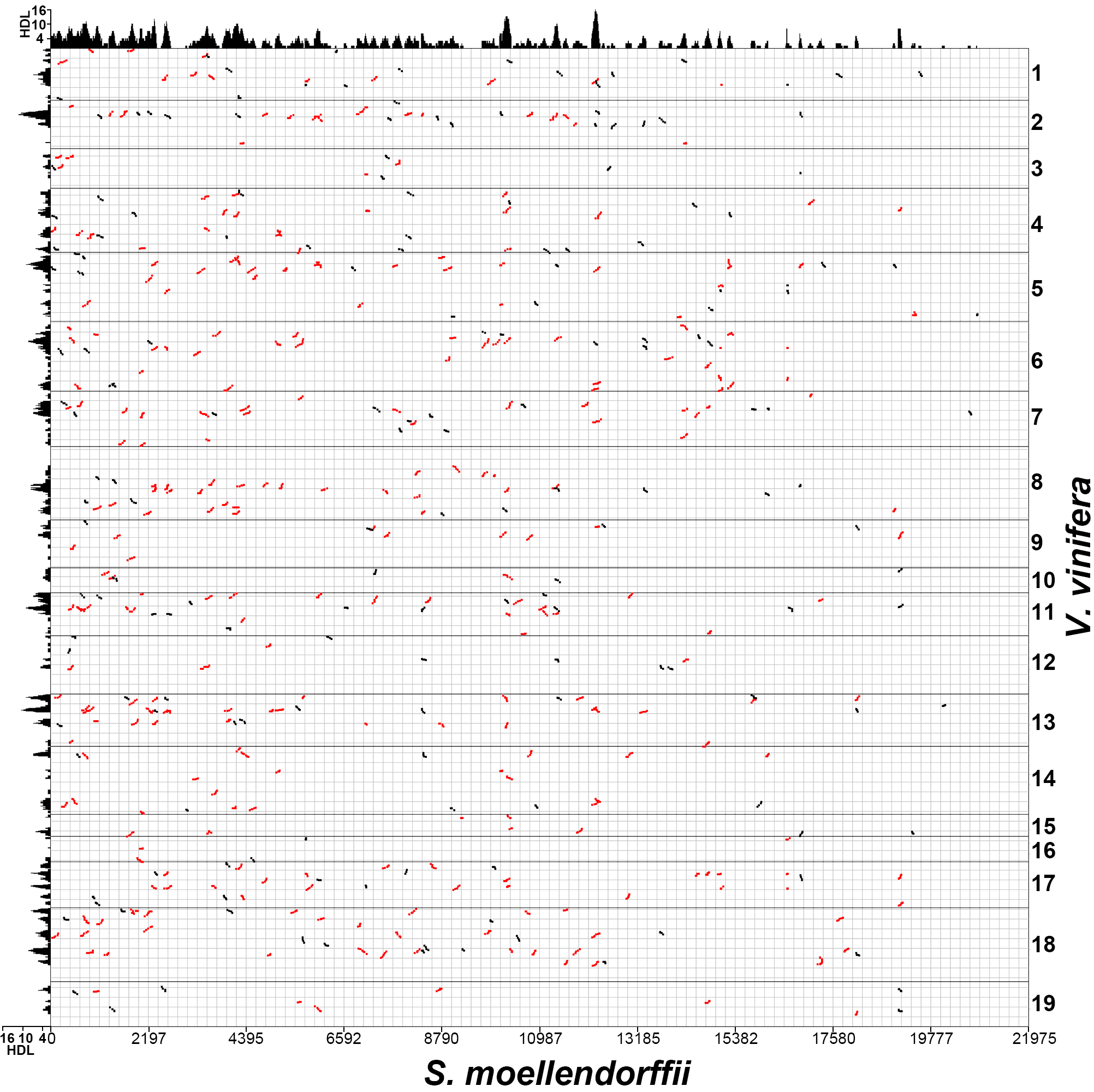

Supplement: Supplementary Figure S5 — Homologous gene dot plot between S. moellendorffii and V. vinifera. Their scaffolds were arranged along X- and Y-axes, respectively. Statistically significant collinear blocks are displayed, and they are mapped onto both axe to produce homologous coverage depth. Scale bars are displayed to the homologous depth level (HDL) in the genome. [file mmc5.zip › Figure S5 Au060120.png]

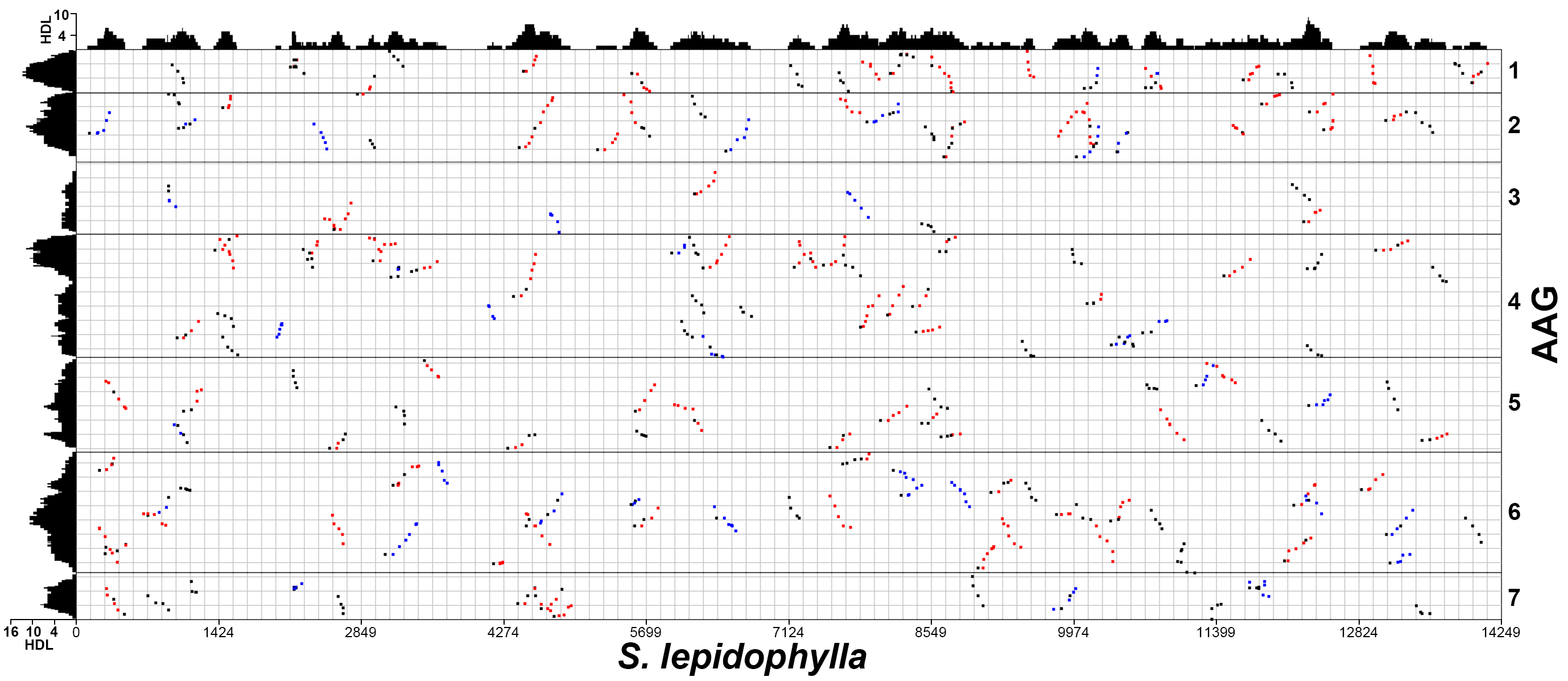

Supplement: Supplementary Figure S6 — Homologous gene dot plot between S. lepidophylla and ancestral angiosperm genomes, i.e., AAG. S. lepidophylla genes and inferred ancestral angiosperm regions represented by genes are arranged along X- and Y-axes, respectively. Statistically significant colinear blocks are displayed and mapped onto each axis to produce homologous coverage depth. Scale bars are displayed to the homologous depth level (HDL) in the genome. Blocks with median Ks < 1.8 are colored red, blocks with Ks ≥ 1.8 colored blue, and others colored gray. [file mmc6.zip › Figure S6 Au060120.png]

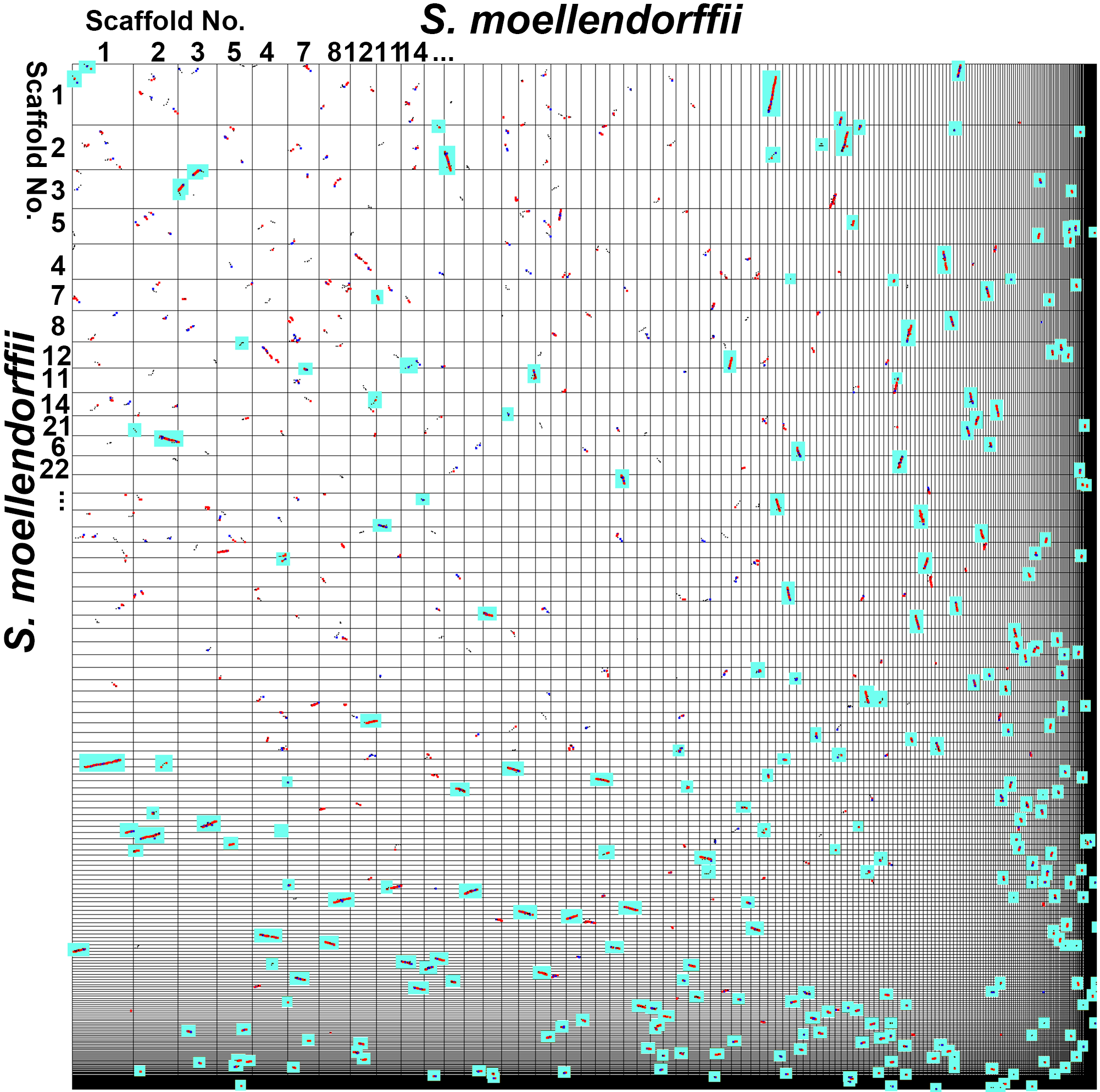

Supplement: Supplementary Figure S7 — Homologous gene dot plot of S. moellendorffii. Blocks that come to the ends of assembled scaffolds are highlighted in blue, details in Figure S1. [file mmc7.zip › Figure S7 Au060120.png]

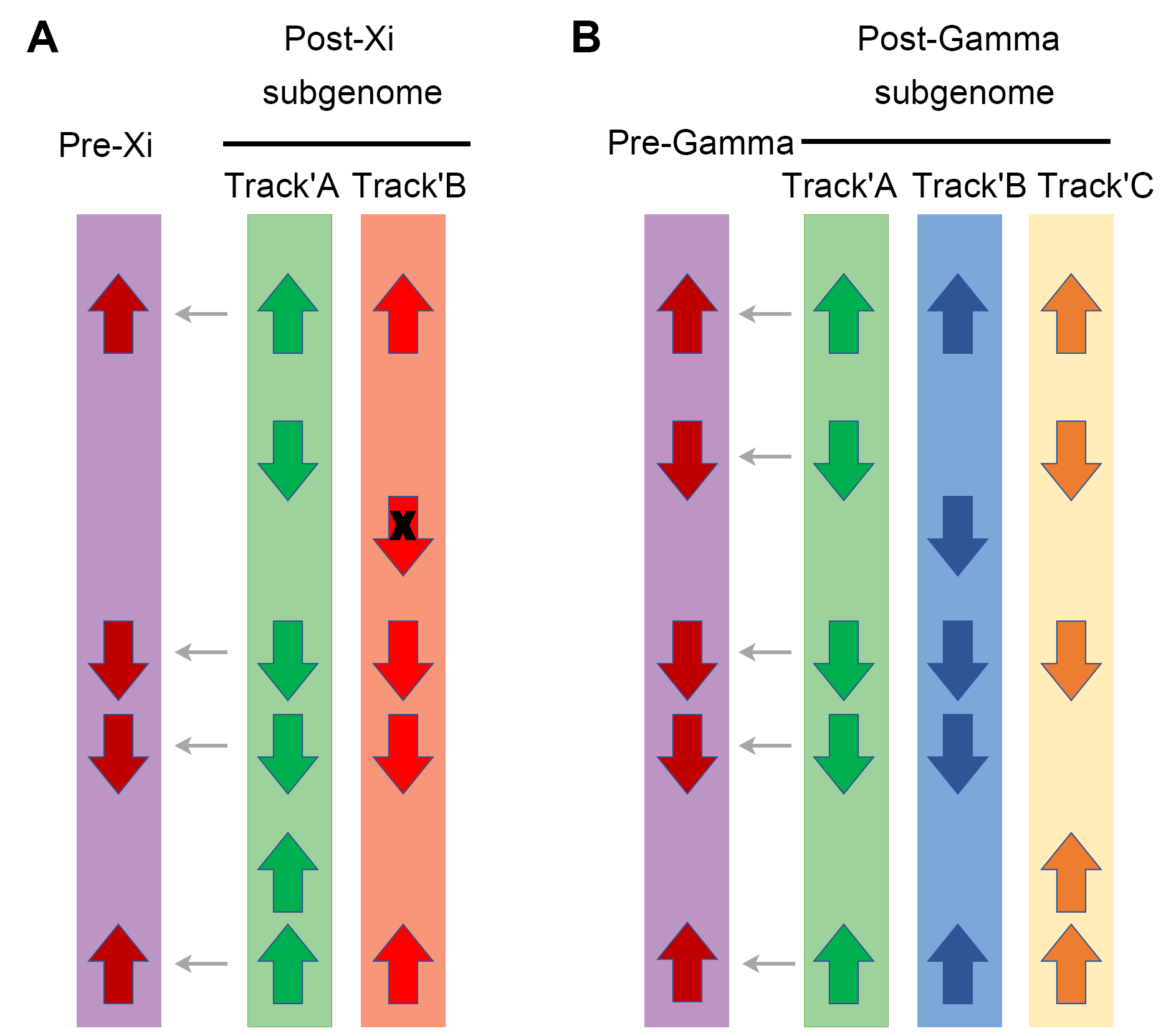

Supplement: Supplementary Figure S8 — Inference of ancestral genomes in Lycophytes and angiosperm lineages. A. The pre-ξ ancestral genome of lycophytes is inferred by merging collinear S. moellendorffii genes produced by ξ. B. The ancestral genome of angiosperms is inferred by merging collinear grape genes produced by the major-eudicot-common hexaploidy. [file mmc8.zip › Figure S8 Au060120.png]
