# Supplementary material for: Paleo-polyploidization in Lycophytes
Source: Genomics Proteomics Bioinformatics. 2020 Nov 4;18(3):333–40. doi: 10.1016/j.gpb.2020.10.002 (PMC7801247; doi:10.1016/j.gpb.2020.10.002)
Supplement: Supplementary Table S1 — Number of colinear blocks and gene pairs within a genome or between genomes. [file mmc9.docx]

**Table S1 Number of colinear blocks and gene pairs within a genome or between genomes**

| **Syntenic blocks within and among genomes** | **BL > 4** | **BL > 10** | **BL > 20** | **BL > 50** | **ACGB** | **LDB^c^** | **LDB on chromosomes** |
| --- | --- | --- | --- | --- | --- | --- | --- |
| *S. moellendorffii* | 2632/302 | 1433/68 | 876/27 | 234/4 | 8.72, 21.07, 33.69, 58.5 | 71 | Sm1-Sm9 |
| *S. lepidophylla* | 538/110 | 10/1 | NF | NF | 4.89, 10.00, -, - | 10 | Sl0001-Sl0010 |
| *S. moellendorffii Recent* *polyploidization* | 1699/116 | 1389/63 | 906/27 | 234/4 | 14.65, 22.05, 33.56, 58.5 | 71 | Sm1-Sm9 |
| *S. moellendorffii ancestral polyploidization* | 567/120 | 12/1 | NF | NF | 4.72, 12, -, - | 12 | Sm4-Sm12 |
| *A. trichopoda* | 422/84 | 11/1 | NF | NF | 5.02, 11, -, - | 11 | Am4-Am78 |
| *S. moellendroffii vs*. *S. lepidophylla* | 11,087/1121 | 6455/224 | 4912/107 | 2604/24 | 9.89, 28.82, 45.91, 108.50 | 152 | Sl0004-Sm4 |
| *V. vinifera vs. S. moellendorffii* | 2801/586 | 12/1 | NF | NF | 4.78, 12, -, - | 12 | Vv17-Sm3 |
| *S. lepidophylla* *vs*. *V. vinifera ancestor* | 1049/203 | 20/2 | NF | NF | 5.17, 10.00, -, - | 10 | Sl1-Vv2 |
| *V. vinifera vs. S. moellendorffii ancestor* | 4513/923 | 12/1 | NF | NF | 4.89, 12, -, - | 12 | Sm2-Vv6 |
| *S. moellendorffii ancestor vs. V. vinifera ancestor* | 881/181 | 11/1 | NF | NF | 4.87, 11, -, - | 11 | Sm7-Vv4 |
| *A. trichopoda vs.* *S. moellendorffii* | 1950/420 | NF | NF | NF | 4.64, -, -, - | 9 | Am47-Sm13 |
| *A. trichopoda vs. V. vinifera ancestor* | 1522/256 | 275/18 | 46/2 | NF | 5.95, 15.28, 23, - | 24 | Am2-Vv3 |
| *S. moellendorffii ancestor vs. V. vinifera ancestor* | 758/153 | 10/1 | NF | NF | 4.95, 10, -, - | 10 | Sm7-Vv4 |
| *S. moellendorffii ancestor vs. A. trichopoda* | 1865/399 | NF | NF | NF | 4.67, -, -, - | 9 | Sm13-Am47 |

*Note*: BL, block_length; ACGP, average colinear gene pairs respectively per block; LDB, number of colinear gene pairs reside in longest duplicated block.
