# Supplementary material for: Paleo-polyploidization in Lycophytes
Source: Genomics Proteomics Bioinformatics. 2020 Nov 4;18(3):333–40. doi: 10.1016/j.gpb.2020.10.002 (PMC7801247; doi:10.1016/j.gpb.2020.10.002)
Supplement: Supplementary Table S2 — Gene number of syntenic blocks within genome or between genomes. [file mmc10.docx]

**Table S2 Gene number of syntenic blocks within genome or between genomes**

| **Syntenic blocks within and among genomes** | **BL > 4** | **BL > 10** | **BL > 20** | **BL > 50** | **LDB** | **LDB on chromosomes** |
| --- | --- | --- | --- | --- | --- | --- |
| *S. moellendorffii* | 4699 | 2854 | 1746 | 466 | 71 | Sm1-Sm9 |
| *S. lepidophylla* | 947 | 20 | NF | NF | 10 | Sl0001-Sl0010 |
| *S. moellendorffii Recent* *polyploidization* | 3258 | 2707 | 1746 | 466 | 71 | Sm1-Sm9 |
| *S. moellendorffii ancestral polyploidization* | 936 | 24 | NF | NF | 12 | Sm4-Sm12 |
| *A. trichopoda* | 782 | 21 | NF | NF | 11 | Am4-Am78 |
| *S. moellendroffii vs*. *S. lepidophylla* | 4560 *vs.* 4811 | 2350 *vs*. 2378 | 1573 *vs*. 1580 | NF | 152 | Sl0004-Sm4 |
| *V. vinifera vs. S. moellendorffii* | 2022 *vs*. 1883 | 12 *vs*. 12 | NF | NF | 12 | Vv17-Sm3 |
| *S. lepidophylla* *vs*. *V. vinifera ancestor* | 723 *vs*. 540 | 20 *vs*. 19 | NF | NF | 10 | Sl1-Vv2 |
| *V. vinifera vs. S. moellendorffii ancestor* | 2615 *vs*. 3179 | 12 *vs*. 12 | NF | NF | 12 | Sm2-Vv6 |
| *S. moellendorffii ancestor vs. V. vinifera ancestor* | 722 *vs*. 492 | 11 *vs*. 11 | NF | NF | 11 | Sm7-Vv4 |
| *A. trichopoda vs. S. moellendorffii* | 1464 *vs*. 1448 | NF | NF | NF | 9 | Am47-Sm13 |
| *A. trichopoda vs. V. vinifera ancestor* | 1275 *vs*. 924 | 275 *vs*. 269 | 46 *vs*. 46 | NF | 24 | Am2-Vv3 |
| *S. moellendorffii ancestor vs. V. vinifera ancestor* | 619 *vs*. 442 | 10 *vs*. 9 | NF | NF | 10 | Sm7-Vv4 |
| *S. moellendorffii ancestor vs. A. trichopoda* | 1312 *vs*. 1396 | NF | NF | NF | 9 | Sm13-Am47 |

*Note*: BL, block_length; LDB, number of colinear gene pairs reside in longest duplicated block.
