# Supplementary material for: Paleo-polyploidization in Lycophytes
Source: Genomics Proteomics Bioinformatics. 2020 Nov 4;18(3):333–40. doi: 10.1016/j.gpb.2020.10.002 (PMC7801247; doi:10.1016/j.gpb.2020.10.002)
Supplement: Supplementary Table S3 — Block information. [file mmc11.docx]

| **Table S3 Block information** | | | | | |
| --- | --- | --- | --- | --- | --- |
| **Block number** | **Scaf1** | **starting-end** | **Scaf2** | **starting-end** | **Block length** |
| 1 | 101 | 551901-602007 | 168 | 11478-130539 | 5 |
| 2 | 102 | 193331-268789 | 105 | 25670-295678 | 7 |
| 3 | 102 | 227340-601531 | 127 | 75654-446863 | 8 |
| 4 | 102 | 256160-495591 | 184 | 10568-111806 | 5 |
| 5 | 105 | 25670-260621 | 127 | 240233-446863 | 5 |
| 6 | 106 | 117972-359845 | 137 | 84038-301052 | 9 |
| 7 | 10 | 2716925-2991902 | 11 | 2689483-2906828 | 5 |
| 8 | 10 | 2567549-2817457 | 135 | 55297-344670 | 10 |
| 9 | 10 | 2799458-3017857 | 139 | 113364-333925 | 7 |
| 10 | 10 | 114901-956373 | 51 | 349494-1264955 | 17 |
| 11 | 115 | 12425-471630 | 123 | 41790-451960 | 11 |
| 12 | 117 | 34920-381362 | 118 | 134850-380195 | 4 |
| 13 | 117 | 64304-387991 | 188 | 79-85179 | 5 |
| 14 | 119 | 386086-401027 | 186 | 34938-75824 | 5 |
| 15 | 11 | 2294481-2715180 | 12 | 1433001-1690983 | 4 |
| 16 | 11 | 421804-1476097 | 15 | 1703573-2744894 | 25 |
| 17 | 11 | 1686844-3035193 | 30 | 331752-1482735 | 30 |
| 18 | 11 | 1475539-1566966 | 30 | 1800800-1874488 | 7 |
| 19 | 11 | 502819-609800 | 40 | 1222195-1495027 | 4 |
| 20 | 11 | 2319962-2970305 | 83 | 153235-790255 | 5 |
| 21 | 120 | 303322-403980 | 175 | 42989-131106 | 4 |
| 22 | 129 | 29301-134909 | 159 | 31537-226111 | 5 |
| 23 | 12 | 259245-757298 | 103 | 199719-581836 | 9 |
| 24 | 12 | 1002071-1380711 | 111 | 194905-571871 | 10 |
| 25 | 12 | 1187587-1657476 | 144 | 84511-284690 | 9 |
| 26 | 12 | 2004769-2510713 | 14 | 1901882-2192992 | 5 |
| 27 | 12 | 2367081-2832457 | 14 | 675175-1554854 | 5 |
| 28 | 12 | 2630124-2711031 | 21 | 1902562-2083110 | 5 |
| 29 | 12 | 1504574-2823569 | 24 | 1057263-1966011 | 22 |
| 30 | 12 | 166837-696545 | 24 | 266979-452750 | 4 |
| 31 | 12 | 1100496-1452534 | 25 | 744359-847266 | 5 |
| 32 | 12 | 1566185-2368527 | 39 | 724452-1536728 | 6 |
| 33 | 12 | 2315287-2704145 | 39 | 674762-822552 | 4 |
| 34 | 12 | 702097-1082007 | 56 | 194235-349265 | 4 |
| 35 | 12 | 1940046-2291755 | 88 | 665232-800617 | 4 |
| 36 | 13 | 1087853-1399638 | 14 | 2373363-2848483 | 4 |
| 37 | 13 | 1228666-2892253 | 16 | 192065-1893851 | 28 |
| 38 | 13 | 2030169-2401663 | 41 | 330899-1044212 | 5 |
| 39 | 13 | 1828841-2473244 | 60 | 473022-1148458 | 4 |
| 40 | 13 | 2216780-2476701 | 65 | 818481-868034 | 5 |
| 41 | 14 | 9745-1410080 | 49 | 154166-1462918 | 21 |
| 42 | 14 | 1673253-2838829 | 67 | 69901-1057946 | 16 |
| 43 | 15 | 75231-1627149 | 38 | 75363-1615084 | 31 |
| 44 | 16 | 1961292-2406149 | 116 | 260398-478437 | 5 |
| 45 | 17 | 159358-1745777 | 53 | 792114-964087 | 6 |
| 46 | 18 | 1420660-1784665 | 122 | 27953-404371 | 9 |
| 47 | 18 | 1133913-1394506 | 173 | 2047-153820 | 6 |
| 48 | 18 | 1126667-1394506 | 28 | 494762-1433844 | 10 |
| 49 | 18 | 156962-589995 | 37 | 649238-823621 | 4 |
| 50 | 19 | 1155172-1306981 | 148 | 183133-296660 | 6 |
| 51 | 19 | 1034769-1164449 | 164 | 6659-148281 | 5 |
| 52 | 19 | 26585-1017782 | 20 | 1366250-2302465 | 27 |
| 53 | 19 | 1455042-2194964 | 35 | 37752-645620 | 15 |
| 54 | 1 | 5609996-6089608 | 10 | 1255456-2890169 | 4 |
| 55 | 1 | 3007291-3398431 | 12 | 2296155-2564850 | 4 |
| 56 | 1 | 5876128-5946239 | 13 | 672007-728286 | 4 |
| 57 | 1 | 2475759-3183248 | 14 | 1624122-2186365 | 6 |
| 58 | 1 | 4462372-4623318 | 14 | 626773-1054797 | 5 |
| 59 | 1 | 5829459-6584283 | 14 | 1129831-1617666 | 5 |
| 60 | 1 | 2031493-2322868 | 15 | 436041-598615 | 5 |
| 61 | 1 | 6557916-6913558 | 15 | 333032-917199 | 5 |
| 62 | 1 | 5149-611454 | 1 | 1586869-2478147 | 4 |
| 63 | 1 | 1586869-2478147 | 1 | 5149-611454 | 4 |
| 64 | 1 | 5465581-5805722 | 20 | 345212-607768 | 6 |
| 65 | 1 | 4997795-5198597 | 20 | 804209-887564 | 5 |
| 66 | 1 | 542619-1509943 | 25 | 955753-1538768 | 5 |
| 67 | 1 | 2650699-3183248 | 25 | 921055-1408802 | 5 |
| 68 | 1 | 4750073-5082041 | 25 | 1537562-1896336 | 4 |
| 69 | 1 | 3402804-3573210 | 2 | 1189916-1757553 | 7 |
| 70 | 1 | 1572673-2140034 | 2 | 3115571-3407496 | 6 |
| 71 | 1 | 1359445-1863304 | 2 | 3008043-3390496 | 5 |
| 72 | 1 | 3930534-4669289 | 2 | 4829422-5080223 | 5 |
| 73 | 1 | 3866798-4199340 | 2 | 2704045-3011928 | 4 |
| 74 | 1 | 5055751-5475718 | 2 | 5518300-6048311 | 4 |
| 75 | 1 | 5829459-6047890 | 36 | 30650-598601 | 6 |
| 76 | 1 | 395200-1134739 | 3 | 1862034-2350664 | 5 |
| 77 | 1 | 616356-1134739 | 3 | 243767-549827 | 4 |
| 78 | 1 | 4652959-4920399 | 43 | 468869-1161380 | 4 |
| 79 | 1 | 3043875-3349363 | 48 | 495895-915557 | 4 |
| 80 | 1 | 241907-411772 | 4 | 409141-999266 | 5 |
| 81 | 1 | 6046582-6904635 | 58 | 460685-1278305 | 16 |
| 82 | 1 | 257179-396207 | 5 | 2280305-2890942 | 4 |
| 83 | 1 | 261775-1594861 | 61 | 32620-1156231 | 29 |
| 84 | 1 | 4171647-4531367 | 6 | 2510229-2631390 | 5 |
| 85 | 1 | 304558-870135 | 7 | 2163227-2785314 | 5 |
| 86 | 1 | 6656260-6702936 | 84 | 669356-719290 | 4 |
| 87 | 1 | 2240090-3010678 | 8 | 2946553-3452196 | 6 |
| 88 | 1 | 4723958-5004255 | 8 | 224207-708805 | 5 |
| 89 | 1 | 1589432-5292107 | 9 | 14894-3648023 | 71 |
| 90 | 20 | 852984-1089194 | 21 | 1248749-1574444 | 4 |
| 91 | 20 | 848137-887564 | 25 | 897934-1270225 | 5 |
| 92 | 20 | 848137-1490767 | 25 | 1269476-1795887 | 4 |
| 93 | 20 | 195020-1026511 | 62 | 481921-1248458 | 18 |
| 94 | 21 | 936966-1183346 | 155 | 23269-236547 | 5 |
| 95 | 21 | 800466-1184241 | 25 | 744359-906144 | 4 |
| 96 | 21 | 801804-1369783 | 29 | 43358-289509 | 5 |
| 97 | 21 | 1064158-1184241 | 30 | 1724647-1801630 | 5 |
| 98 | 21 | 428964-691332 | 39 | 651036-1147081 | 4 |
| 99 | 21 | 145296-616259 | 39 | 827288-1296993 | 4 |
| 100 | 21 | 1563795-2087573 | 41 | 1045789-1285345 | 5 |
| 101 | 21 | 1180879-2004762 | 63 | 21777-782663 | 17 |
| 102 | 21 | 65637-827871 | 87 | 92743-814798 | 28 |
| 103 | 22 | 1435110-1869621 | 166 | 25283-209174 | 11 |
| 104 | 22 | 41308-1437480 | 32 | 50922-1801810 | 42 |
| 105 | 22 | 366509-762048 | 44 | 854631-1301978 | 4 |
| 106 | 22 | 1330659-1581942 | 52 | 187332-366311 | 4 |
| 107 | 23 | 37070-1621623 | 29 | 475858-1908053 | 25 |
| 108 | 23 | 1003802-1559838 | 40 | 163018-1304947 | 6 |
| 109 | 23 | 1063949-1589007 | 47 | 990842-1394433 | 5 |
| 110 | 23 | 961365-1182678 | 86 | 143648-455599 | 4 |
| 111 | 24 | 59219-798633 | 25 | 897934-1454364 | 4 |
| 112 | 24 | 43402-502584 | 40 | 402840-1418912 | 7 |
| 113 | 24 | 38182-811130 | 59 | 192458-1031398 | 14 |
| 114 | 25 | 1541667-1896336 | 113 | 83509-492342 | 9 |
| 115 | 25 | 1544208-1842389 | 33 | 269009-682614 | 4 |
| 116 | 25 | 475953-1450029 | 70 | 24553-1023696 | 20 |
| 117 | 27 | 338445-1587691 | 35 | 344231-1757659 | 18 |
| 118 | 27 | 604486-1131006 | 60 | 572110-675295 | 5 |
| 119 | 27 | 57342-458914 | 93 | 100385-696390 | 7 |
| 120 | 28 | 925561-1433844 | 149 | 70424-267170 | 4 |
| 121 | 28 | 349668-730103 | 158 | 25923-235053 | 6 |
| 122 | 28 | 449476-926298 | 173 | 2047-153820 | 8 |
| 123 | 28 | 510756-1433844 | 173 | 2047-153820 | 7 |
| 124 | 28 | 9411-441612 | 82 | 466-279376 | 5 |
| 125 | 29 | 318753-462787 | 162 | 3680-186569 | 5 |
| 126 | 29 | 822-294678 | 39 | 1289028-1624497 | 8 |
| 127 | 2 | 1204550-1363444 | 107 | 123123-391829 | 4 |
| 128 | 2 | 2647270-3210306 | 11 | 452889-942749 | 5 |
| 129 | 2 | 719710-1379079 | 13 | 811584-1103697 | 5 |
| 130 | 2 | 502590-1150129 | 14 | 2373363-2801297 | 5 |
| 131 | 2 | 5441822-6055017 | 14 | 1609428-1875850 | 5 |
| 132 | 2 | 1360511-3895326 | 17 | 117754-2463304 | 51 |
| 133 | 2 | 60410-565324 | 21 | 1296271-1660617 | 4 |
| 134 | 2 | 5622268-6055017 | 22 | 366509-580713 | 6 |
| 135 | 2 | 1379279-1675124 | 23 | 613141-975013 | 4 |
| 136 | 2 | 71576-240341 | 23 | 1017230-1311701 | 4 |
| 137 | 2 | 4980267-5328372 | 25 | 1416951-1547393 | 5 |
| 138 | 2 | 1874433-2445541 | 25 | 1676693-1916293 | 4 |
| 139 | 2 | 62504-865739 | 47 | 59300-787592 | 12 |
| 140 | 2 | 3053381-3288894 | 53 | 949628-1300922 | 5 |
| 141 | 2 | 3053381-3517482 | 53 | 902099-1043518 | 4 |
| 142 | 2 | 2092172-2423092 | 5 | 629182-932024 | 6 |
| 143 | 2 | 478349-1150129 | 5 | 1358141-1618931 | 4 |
| 144 | 2 | 2628228-3115232 | 5 | 334904-494781 | 4 |
| 145 | 2 | 5082079-5239789 | 5 | 2601934-2643634 | 4 |
| 146 | 2 | 498439-1054408 | 62 | 108208-1232582 | 5 |
| 147 | 2 | 4060985-6166316 | 6 | 66431-2378985 | 57 |
| 148 | 2 | 3482679-3816272 | 7 | 358478-634049 | 5 |
| 149 | 2 | 3117890-3484391 | 7 | 1136868-1566751 | 4 |
| 150 | 2 | 4375529-4981968 | 8 | 706857-1178327 | 5 |
| 151 | 2 | 4114924-4760704 | 9 | 1483705-3646253 | 4 |
| 152 | 31 | 604462-889303 | 115 | 196880-252984 | 5 |
| 153 | 31 | 938339-1037777 | 71 | 26853-253095 | 4 |
| 154 | 31 | 66720-784133 | 80 | 184748-795954 | 13 |
| 155 | 33 | 767256-1814398 | 115 | 97958-332810 | 6 |
| 156 | 33 | 582-1785366 | 46 | 117826-1498799 | 31 |
| 157 | 34 | 946822-1225590 | 130 | 52-264907 | 10 |
| 158 | 36 | 4365-1296338 | 42 | 58907-1626375 | 30 |
| 159 | 36 | 479757-815293 | 65 | 292323-761688 | 5 |
| 160 | 36 | 262291-639537 | 65 | 306629-743489 | 5 |
| 161 | 36 | 1221319-1725225 | 78 | 394178-814334 | 7 |
| 162 | 39 | 617230-1121901 | 58 | 221119-1278305 | 5 |
| 163 | 39 | 159419-773417 | 64 | 381793-804664 | 8 |
| 164 | 39 | 617230-780804 | 88 | 659174-800617 | 4 |
| 165 | 3 | 1903749-2237667 | 10 | 613894-1187668 | 6 |
| 166 | 3 | 4128995-4327356 | 12 | 1965931-2331134 | 5 |
| 167 | 3 | 3032656-3531451 | 13 | 2527437-2770068 | 5 |
| 168 | 3 | 2423134-2565771 | 142 | 58187-313209 | 5 |
| 169 | 3 | 15870-365407 | 3 | 2347143-2886186 | 5 |
| 170 | 3 | 2347143-2886186 | 3 | 15870-365407 | 5 |
| 171 | 3 | 3171595-3725493 | 44 | 775121-1127180 | 4 |
| 172 | 3 | 2667406-4309215 | 45 | 88793-1532771 | 55 |
| 173 | 3 | 3032656-3535974 | 6 | 3638058-3909192 | 4 |
| 174 | 3 | 559748-902129 | 7 | 2373505-2489710 | 4 |
| 175 | 3 | 983215-1930860 | 8 | 1979838-2383208 | 7 |
| 176 | 3 | 1097938-1464782 | 98 | 4782-446296 | 16 |
| 177 | 40 | 646387-1066352 | 121 | 76865-458565 | 15 |
| 178 | 40 | 15373-766345 | 65 | 590998-979896 | 6 |
| 179 | 40 | 1554537-1707764 | 74 | 750442-897521 | 8 |
| 180 | 40 | 990333-1215154 | 75 | 678-201068 | 5 |
| 181 | 40 | 1148082-1328594 | 83 | 153235-531976 | 4 |
| 182 | 40 | 28516-353055 | 89 | 522077-695482 | 4 |
| 183 | 41 | 1275097-1690080 | 108 | 97781-533982 | 10 |
| 184 | 41 | 61996-903534 | 90 | 20586-766487 | 18 |
| 185 | 43 | 105722-894095 | 68 | 270808-1056845 | 17 |
| 186 | 43 | 1159083-1528608 | 89 | 80034-432780 | 17 |
| 187 | 44 | 827253-1234301 | 126 | 35187-417633 | 11 |
| 188 | 44 | 595666-784022 | 140 | 7350-315938 | 9 |
| 189 | 44 | 1185711-1518780 | 145 | 24769-301304 | 7 |
| 190 | 44 | 1572046-1643413 | 174 | 53485-167457 | 7 |
| 191 | 44 | 59565-614454 | 82 | 304448-889490 | 10 |
| 192 | 44 | 365599-850461 | 82 | 602587-889490 | 5 |
| 193 | 46 | 1407764-1489952 | 115 | 12425-139451 | 6 |
| 194 | 46 | 599046-1500941 | 115 | 130434-329439 | 6 |
| 195 | 47 | 995177-1504360 | 97 | 5923-600209 | 8 |
| 196 | 48 | 73836-1415181 | 55 | 22715-1263822 | 34 |
| 197 | 4 | 3481358-4056812 | 10 | 2731565-2990601 | 5 |
| 198 | 4 | 1583610-2670065 | 12 | 557880-1684391 | 15 |
| 199 | 4 | 3096387-3278406 | 12 | 1906880-2227326 | 4 |
| 200 | 4 | 3396220-3752615 | 15 | 1024217-1444139 | 4 |
| 201 | 4 | 3579891-3795793 | 18 | 2206466-2420611 | 7 |
| 202 | 4 | 3643448-3996963 | 21 | 1568765-2084340 | 5 |
| 203 | 4 | 989956-1448667 | 23 | 1394403-1917650 | 4 |
| 204 | 4 | 479887-999266 | 23 | 206631-431266 | 4 |
| 205 | 4 | 3880384-4129450 | 24 | 380535-461406 | 5 |
| 206 | 4 | 1354104-3234658 | 26 | 71861-1864104 | 37 |
| 207 | 4 | 858413-1764295 | 33 | 744837-1623768 | 6 |
| 208 | 4 | 3786316-4051045 | 36 | 81452-357725 | 4 |
| 209 | 4 | 436635-1308935 | 50 | 394204-1181690 | 11 |
| 210 | 4 | 2237011-2322390 | 52 | 796872-1147281 | 4 |
| 211 | 4 | 1222459-1532169 | 5 | 1616484-2036661 | 5 |
| 212 | 4 | 2670357-2940188 | 5 | 1295109-1420387 | 4 |
| 213 | 4 | 3409258-4132465 | 65 | 420550-822193 | 6 |
| 214 | 4 | 2704676-2807864 | 68 | 12144-484282 | 4 |
| 215 | 4 | 4286069-4337861 | 74 | 268022-280884 | 4 |
| 216 | 4 | 2019636-2518101 | 7 | 2544331-2964383 | 5 |
| 217 | 4 | 4286069-4340302 | 91 | 371059-426125 | 5 |
| 218 | 52 | 77311-735860 | 56 | 542308-1225110 | 10 |
| 219 | 52 | 865899-1175175 | 72 | 310-580321 | 11 |
| 220 | 53 | 762118-1068768 | 131 | 85314-398752 | 14 |
| 221 | 56 | 111667-502642 | 114 | 18302-501941 | 12 |
| 222 | 58 | 143888-1076178 | 60 | 364500-834604 | 4 |
| 223 | 58 | 2011-40544 | 83 | 830740-851715 | 5 |
| 224 | 58 | 6126-265036 | 84 | 279856-487664 | 6 |
| 225 | 59 | 1082186-1248517 | 117 | 64304-412989 | 5 |
| 226 | 59 | 1082186-1138623 | 188 | 79-71495 | 4 |
| 227 | 5 | 55890-998523 | 10 | 1306906-2521751 | 17 |
| 228 | 5 | 1676964-2172689 | 128 | 18399-350889 | 10 |
| 229 | 5 | 2231411-2848505 | 12 | 90173-296022 | 5 |
| 230 | 5 | 1813411-2305824 | 150 | 11631-250300 | 9 |
| 231 | 5 | 18544-266386 | 15 | 902477-1384613 | 5 |
| 232 | 5 | 434360-777142 | 15 | 322409-636584 | 4 |
| 233 | 5 | 2371476-2433353 | 198 | 13315-67112 | 5 |
| 234 | 5 | 2687606-3543237 | 21 | 183114-536479 | 5 |
| 235 | 5 | 1250510-1718157 | 25 | 1209315-1815900 | 4 |
| 236 | 5 | 2601934-2932371 | 25 | 1537562-1901323 | 4 |
| 237 | 5 | 1218727-1590237 | 28 | 1562463-1949409 | 6 |
| 238 | 5 | 208661-340124 | 40 | 1308985-1546445 | 4 |
| 239 | 5 | 3268106-3924084 | 7 | 432016-982810 | 5 |
| 240 | 5 | 2630751-3271853 | 92 | 10826-647008 | 9 |
| 241 | 5 | 3504007-3838167 | 99 | 176231-541547 | 8 |
| 242 | 60 | 246595-641882 | 110 | 100456-434843 | 11 |
| 243 | 60 | 823956-1124445 | 94 | 136794-371131 | 4 |
| 244 | 62 | 145161-232989 | 129 | 702-166625 | 4 |
| 245 | 62 | 86643-224927 | 159 | 31537-203176 | 5 |
| 246 | 65 | 6764-852396 | 71 | 201175-982415 | 34 |
| 247 | 66 | 53197-90718 | 100 | 431729-444528 | 4 |
| 248 | 66 | 170749-688871 | 69 | 318483-437685 | 4 |
| 249 | 66 | 53197-902883 | 73 | 32189-957272 | 19 |
| 250 | 69 | 433185-939258 | 102 | 94775-495591 | 4 |
| 251 | 69 | 318483-939258 | 79 | 36791-626403 | 11 |
| 252 | 6 | 2057837-3044015 | 22 | 1330659-1940311 | 6 |
| 253 | 6 | 3034800-3547479 | 27 | 190783-775024 | 4 |
| 254 | 6 | 2985314-4103504 | 34 | 57563-963511 | 29 |
| 255 | 6 | 2534641-2917536 | 75 | 453075-967354 | 13 |
| 256 | 71 | 189579-268530 | 266 | 848-16095 | 5 |
| 257 | 72 | 578012-770920 | 100 | 244424-543882 | 7 |
| 258 | 73 | 32189-68124 | 100 | 431729-445277 | 4 |
| 259 | 74 | 225265-748041 | 91 | 48688-454613 | 12 |
| 260 | 76 | 160966-554928 | 112 | 79279-524276 | 5 |
| 261 | 79 | 36791-191728 | 102 | 235605-495591 | 6 |
| 262 | 79 | 36791-191728 | 105 | 27975-167671 | 4 |
| 263 | 79 | 57128-191728 | 184 | 10568-111806 | 4 |
| 264 | 7 | 2040442-2548557 | 11 | 91-419866 | 15 |
| 265 | 7 | 803570-1286556 | 11 | 1686844-1952666 | 5 |
| 266 | 7 | 799920-1450538 | 11 | 1648090-1952666 | 5 |
| 267 | 7 | 799920-1265557 | 13 | 1036461-1797604 | 4 |
| 268 | 7 | 2164753-2769670 | 14 | 700475-1120473 | 5 |
| 269 | 7 | 631018-853619 | 151 | 31724-272165 | 10 |
| 270 | 7 | 2981702-3919867 | 18 | 117477-754929 | 21 |
| 271 | 7 | 8874-185005 | 30 | 85838-318949 | 5 |
| 272 | 7 | 782369-1101090 | 37 | 59877-766771 | 4 |
| 273 | 7 | 928100-1302582 | 48 | 94652-354556 | 4 |
| 274 | 7 | 881613-2099167 | 57 | 10602-1297103 | 31 |
| 275 | 7 | 171213-972641 | 8 | 3136602-3673432 | 7 |
| 276 | 7 | 318319-490777 | 8 | 1014425-1259636 | 5 |
| 277 | 7 | 799920-1363058 | 8 | 3311778-3697933 | 5 |
| 278 | 7 | 28707-173623 | 8 | 1427687-1559941 | 4 |
| 279 | 7 | 2552914-2764940 | 96 | 558046-694406 | 4 |
| 280 | 81 | 441920-795960 | 95 | 317144-700990 | 4 |
| 281 | 83 | 379346-837032 | 84 | 485503-811523 | 8 |
| 282 | 85 | 421012-824104 | 109 | 39165-475421 | 12 |
| 283 | 86 | 304676-808744 | 119 | 14002-404831 | 12 |
| 284 | 86 | 618891-766473 | 186 | 10-108003 | 8 |
| 285 | 86 | 686622-796009 | 90 | 20586-52369 | 5 |
| 286 | 88 | 580170-815438 | 120 | 45665-311738 | 10 |
| 287 | 8 | 2791772-2988659 | 10 | 239150-758563 | 5 |
| 288 | 8 | 2172524-2930882 | 12 | 166837-523677 | 5 |
| 289 | 8 | 1558387-1568769 | 131 | 74093-81963 | 4 |
| 290 | 8 | 656711-856125 | 13 | 418565-514986 | 5 |
| 291 | 8 | 3045055-3247131 | 18 | 565122-921213 | 8 |
| 292 | 8 | 2639432-2932438 | 19 | 142469-295474 | 4 |
| 293 | 8 | 236333-1018824 | 27 | 664201-1250225 | 5 |
| 294 | 8 | 1958723-3684881 | 37 | 48148-1713842 | 42 |
| 295 | 8 | 2893575-3285963 | 41 | 1092254-1371274 | 5 |
| 296 | 8 | 2042249-2428397 | 41 | 598971-1094816 | 5 |
| 297 | 8 | 2616790-2692162 | 48 | 800279-982194 | 4 |
| 298 | 8 | 18571-693831 | 53 | 220027-706434 | 9 |
| 299 | 8 | 922311-1964501 | 54 | 281847-1418059 | 25 |
| 300 | 8 | 678988-1178327 | 8 | 2377470-2789109 | 4 |
| 301 | 8 | 2377470-2789109 | 8 | 678988-1178327 | 4 |
| 302 | 90 | 20586-52369 | 186 | 40445-105691 | 4 |
