# Supplementary material for: Paleo-polyploidization in Lycophytes
Source: Genomics Proteomics Bioinformatics. 2020 Nov 4;18(3):333–40. doi: 10.1016/j.gpb.2020.10.002 (PMC7801247; doi:10.1016/j.gpb.2020.10.002)
Supplement: Supplementary Table S4 — Homology depth in the S. moellendorffii genome. [file mmc12.docx]

**Table S4 Homology depth in the *S. moellendorffii* genome**

| **Homologous depth level** | ***S. moellendorffii* genes were covered by syntenic blocks with 4 or more colinear genes** |
| --- | --- |
| 0 | 2370 of 21,975 (10.78%) |
| 1 | 5946 of 21,975 (27.04%) |
| 2 | 5573 of 21,975 (25.36%) |
| 3 | 3857 of 21,975 (17.55%) |
| 4 | 2301 of 21,975 (10.47%) |
| 5 | 1308 of 21,975 (5.95%) |
| 6 | 411 of 21,975 (1.87%) |
| 7 | 99 of 21,975 (0.45%) |
| 8 | 64 of 21,975 (0.29%) |
| 9 | 40 of 21,975 (0.18%) |
| 10 | 6 of 21,975 (0.03%) |
