# Supplementary material for: Paleo-polyploidization in Lycophytes
Source: Genomics Proteomics Bioinformatics. 2020 Nov 4;18(3):333–40. doi: 10.1016/j.gpb.2020.10.002 (PMC7801247; doi:10.1016/j.gpb.2020.10.002)
Supplement: Supplementary Table S5 — Homology depth between S. moellendorffii and A. trichopoda genomes. [file mmc13.docx]

**Table S5 Homology depth between *S. moellendorffii* and *A. trichopoda* genomes**

| **Homologous depth level** | ***A. trichopoda* regions aligned to *S. moellendorffii*** | ***S. moellendorffii* regions aligned to *A. trichopoda*** |
| --- | --- | --- |
| 0 | 10,941 of 21,975(49.79%) | 14,437 of 26,846 (53.78%) |
| 1 | 4110 of 21,975(18.70%) | 4635 of 26,846 (17.27%) |
| 2 | 2379 of 21,975(10.83%) | 3200 of 26,846 (11.92%) |
| 3 | 1430 of 21,975(6.51%) | 1960 of 26,846 (7.30%) |
| 4 | 1305 of 21,975(5.94%) | 983 of 26,846 (3.66%) |
| 5 | 787 of 21,975(3.58%) | 478 of 26,846 (1.78%) |
| 6 | 481 of 21,975(2.19%) | 430 of 26,846 (1.60%) |
| 7 | 316 of 21,975(1.44%) | 280 of 26,846 (1.04%) |
| 8 | 106 of 21,975 (0.48%) | 258 of 26,846 (0.96%) |
| 9 | 63 of 21,975 (0.29%) | 115 of 26,846 (0.43%) |
| 10 | 31 of 21,975 (0.14%) | 24 of 26,846 (0.09%) |
| 11 | 6 of 21,975 (0.03%) | 15 of 26,846 (0.06%) |
| 12 | 14 of 21,975 (0.06) | 6 of 26,846 (0.02%) |
| 13 | 6 of 21,975 (0.03%) | 21 of 26,846 (0.08%) |
| 14 |  | 4 of 26,846 (0.01%) |
