# Supplementary material for: Paleo-polyploidization in Lycophytes
Source: Genomics Proteomics Bioinformatics. 2020 Nov 4;18(3):333–40. doi: 10.1016/j.gpb.2020.10.002 (PMC7801247; doi:10.1016/j.gpb.2020.10.002)
Supplement: Supplementary Table S6 — Homology depth between S. moellendorffii and V. vinifera genomes. [file mmc14.docx]

**Table S6 Homology depth between *S. moellendorffii* and *V. vinifera* genomes**

| **Homologous depth level** | ***V. vinifera* regions aligned to *S. moellendorffii*** | ***S. moellendorffii* regions aligned to *V. vinifera*** |
| --- | --- | --- |
| 0 | 8804 of 21,975 (40.06%) | 10577 of 24,901 (42.48%) |
| 1 | 3237 of 21,975 (14.73%) | 5437 of 24,901 (21.83%) |
| 2 | 3107 of 21,975 (14.14%) | 3460 of 24,901 (13.90%) |
| 3 | 2140 of 21,975 (9.74%) | 1975 of 24,901 (7.93%) |
| 4 | 1495 of 21,975 (6.80%) | 1429 of 24,901 (5.74%) |
| 5 | 1012 of 21,975 (4.61%) | 677 of 24,901 (2.72%) |
| 6 | 605 of 21,975 (2.75%) | 435 of 24,901 (1.75%) |
| 7 | 617 of 21,975 (2.81%) | 484 of 24,901 (1.94%) |
| 8 | 333 of 21,975 (1.52%) | 172 of 24,901 (0.69%) |
| 9 | 272 of 21,975 (1.24%) | 132 of 24,901 (0.53%) |
| 10 | 130 of 21,975 (0.59%) | 57 of 24,901 (0.23%) |
| 11 | 108 of 21,975 (0.49%) | 36 of 24,901 (0.14%) |
| 12 | 44 of 21,975 (0.20%) | 24 of 24,901 (0.10%) |
| 13 | 21 of 21,975 (0.10%) | 6 of 24,901 (0.02%) |
| 14 | 21 of 21,975 (0.10%) |  |
| 15 | 28 of 21,975 (0.13%) |  |
| 16 | 1 of 21,975 (0.00%) |  |
