# Supplementary material for: Paleo-polyploidization in Lycophytes
Source: Genomics Proteomics Bioinformatics. 2020 Nov 4;18(3):333–40. doi: 10.1016/j.gpb.2020.10.002 (PMC7801247; doi:10.1016/j.gpb.2020.10.002)
Supplement: Supplementary Table S7 — Homology depth between ALG and AAG genomes. [file mmc15.docx]

**Table S7 Homology depth between ALG and AAG genomes**

| **Homologous depth level** | **AAG regions aligned to ALG** | **ALG regions aligned to AAG** |
| --- | --- | --- |
| 0 | 4773 of 11,509 (41.47%) | 104 of 1686 (6.17%) |
| 1 | 3036 of 11,509 (26.38%) | 75 of 1686 (4.45%) |
| 2 | 1705 of 11,509 (14.81%) | 107 of 1686 (6.35%) |
| 3 | 1132 of 11,509 (9.84%) | 80 of 1686 (4.75%) |
| 4 | 658 of 11,509 (5.72%) | 181 of 1686 (10.74%) |
| 5 | 157 of 11,509 (1.36%) | 139 of 1686 (8.24%) |
| 6 | 43 of 11,509 (0.37%) | 159 of 1686 (9.43%) |
| 7 | 5 of 11,509 (0.04%) | 146 of 1686 (8.66%) |
| 8 |  | 55 of 1686 (3.26%) |
| 9 |  | 103 of 1686 (6.11%) |
| 10 |  | 53 of 1686 (3.14%) |
| 11 |  | 95 of 1686 (5.63%) |
| 12 |  | 114 of 1686 (6.76%) |
| 13 |  | 109 of 1686 (6.46%) |
| 14 |  | 46 of 1686 (2.73%) |
| 15 |  | 47 of 1686 (2.79%) |
| 16 |  | 34 of 1686 (2.02%) |
| 17 |  | 13 of 1686 (0.77%) |
| 18 |  | 26 of 1686 (1.54%) |

*Note*: ALG gap sizes = 50; AAG gap sizes = 50.

| 0 | 6146 of 7302 (84.17%) | 984 of 1686 (58.36%) |
| --- | --- | --- |
| 1 | 977 of 7302 (13.38%) | 433 of 1686 (25.68%) |
| 2 | 140 of 7302 (1.92%) | 122 of 1686 (7.24%) |
| 3 | 39 of 7302 (0.53%) | 48 of 1686 (2.85%) |
| 4 |  | 53 of 1686 (3.14%) |
| 5 |  | 3 of 1686 (0.18%) |
| 6 |  | 6 of 1686 (0.36%) |
| 7 |  | 11 of 1686 (0.65%) |
| 8 |  | 8 of 1686 (0.47%) |
| 9 |  | 18 of 1686 (1.08%) |

*Note*: ALG gap sizes = 30; AAG gap sizes = 30.

| 0 | 6619 of 10,800 (61.29%) | 255 of 1686 (15.12%) |
| --- | --- | --- |
| 1 | 3033 of 10,800 (28.08%) | 296 of 1686 (17.56%) |
| 2 | 837 of 10,800 (7.75%) | 339 of 1686 (20.11%) |
| 3 | 244 of 10,800 (2.26%) | 203 of 1686 (12.04%) |
| 4 | 51 of 10,800 (0.47%) | 164 of 1686 (9.73%) |
| 5 | 16 of 10,800 (0.15%) | 144 of 1686 (8.54%) |
| 6 |  | 80 of 1686 (4.75%) |
| 7 |  | 45 of 1686 (2.67%) |
| 8 |  | 16 of 1686 (0.95%) |
| 9 |  | 45 of 1686 (2.67%) |
| 10 |  | 53 of 1686 (3.14%) |
| 11 |  | 20 of 1686 (1.19%) |
| 12 |  | 9 of 1686 (0.53%) |
| 13 |  | 17 of 1686 (1.01%) |

*Note*: ALG gap sizes = 40; AAG gap sizes = 40.

| 0 | 6207 of 10,605 (58.53%) | 125 of 1686 (7.41%) |
| --- | --- | --- |
| 1 | 2740 of 10,605 (25.84%) | 67 of 1686 (3.97%) |
| 2 | 1022 of 10,605 (9.64%) | 82 of 1686 (4.86%) |
| 3 | 441 of 10,605 (4.16%) | 104 of 1686 (6.17%) |
| 4 | 154 of 10,605 (1.45%) | 106 of 1686 (6.28%) |
| 5 | 41 of 10,605 (0.39%) | 144 of 1686 (8.54%) |
| 6 |  | 98 of 1686 (5.81%) |
| 7 |  | 60 of 1686 (3.56%) |
| 8 |  | 73 of 1686 (4.33%) |
| 9 |  | 134 of 1686 (7.95%) |
| 10 |  | 70 of 1686 (4.15%) |
| 11 |  | 106 of 1686 (6.29%) |
| 12 |  | 53 of 1686 (3.14%) |
| 13 |  | 128 of 1686 (7.59%) |
| 14 |  | 56 of 1686 (3.32%) |
| 15 |  | 42 of 1686 (2.49%) |
| 16 |  | 55 of 1686 (3.26%) |
| 17 |  | 49 of 1686 (2.91%) |
| 18 |  | 52 of 1686 (3.08%) |
| 19 |  | 21 of 1686 (1.25%) |
| 20 |  | 24 of 1686 (1.42%) |
| 21 |  | 15 of 1686 (0.89%) |
| 22 |  | 18 of 1686 (1.06%) |
| 23 |  | 4 of 1686 (0.24%) |

*Note*: ALG gap sizes = 30; AAG gap sizes = 80.

| 0 | 5480 of 11,617 (47.17%) | 65 of 1686 (3.86%) |
| --- | --- | --- |
| 1 | 3245 of 11,617 (27.93%) | 33 of 1686 (1.96%) |
| 2 | 1517 of 11,617 (13.06%) | 23 of 1686 (1.36%) |
| 3 | 738 of 11,617 (6.35%) | 66 of 1686 (3.91%) |
| 4 | 416 of 11,617 (3.58%) | 26 of 1686 (1.54%) |
| 5 | 209 of 11,617 (1.80%) | 47 of 1686 (2.79%) |
| 6 | 12 of 11,617 (0.10%) | 36 of 1686 (2.14%) |
| 7 |  | 64 of 1686 (3.80%) |
| 8 |  | 74 of 1686 (4.39%) |
| 9 |  | 94 of 1686 (5.58%) |
| 10 |  | 85 of 1686 (5.04%) |
| 11 |  | 43 of 1686 (2.55%) |
| 12 |  | 21 of 1686 (1.25%) |
| 13 |  | 21 of 1686 (1.25%) |
| 14 |  | 15 of 1686 (0.89%) |
| 15 |  | 11 of 1686 (0.65%) |
| 16 |  | 24 of 1686 (1.42%) |
| 17 |  | 21 of 1686 (1.25%) |
| 18 |  | 24 of 1686 (1.42%) |
| 19 |  | 26 of 1686 (1.54%) |
| 20 |  | 16 of 1686 (0.95%) |
| 21 |  | 32 of 1686 (1.90%) |
| 22 |  | 57 of 1686 (3.38%) |
| 23 |  | 21 of 1686 (1.25%) |
| 24 |  | 6 of 1686 (0.36%) |
| 25 |  | 16 of 1686 (0.95%) |
| 26 |  | 30 of 1686 (1.78%) |
| 27 |  | 28 of 1686 (1.66%) |
| 28 |  | 28 of 1686 (1.66%) |
| 29 |  | 13 of 1686 (0.77%) |
| 30 |  | 19 of 1686 (1.12%) |
| 31 |  | 28 of 1686 (1.66%) |
| 32 |  | 21 of 1686 (1.25%) |
| 33 |  | 32 of 1686 (1.90%) |
| 34 |  | 28 of 1686 (1.66%) |
| 35 |  | 49 of 1686 (2.91%) |
| 36 |  | 27 of 1686 (1.60%) |
| 37 |  | 17 of 1686 (1.01%) |
| 38 |  | 38 of 1686 (2.25%) |
| 39 |  | 30 of 1686 (1.78%) |
| 40 |  | 29 of 1686 (1.72%) |
| 41 |  | 77 of 1686 (4.57%) |
| 42 |  | 44 of 1686 (2.61%) |
| 43 |  | 13 of 1686 (0.77%) |
| 44 |  | 29 of 1686 (1.72%) |
| 45 |  | 6 of 1686 (0.36%) |
| 46 |  | 29 of 1686 (1.72%) |
| 47 |  | 62 of 1686 (3.68%) |
| 48 |  | 15 of 1686 (0.89%) |
| 49 |  | 26 of 1686 (1.54%) |
| 50 |  | 1 of 1686 (0.06%) |

*Note*: ALG gap sizes = 30; AAG gap sizes = 270.

| 0 | 6784 of 9,068 (74.81%) | 749 of 1686 (44.42%) |
| --- | --- | --- |
| 1 | 1645 of 9,068 (18.14%) | 451 of 1686 (26.75%) |
| 2 | 523 of 9,068 (5.77%) | 215 of 1686 (12.775%) |
| 3 | 116 of 9,068 (1.28%) | 95 of 1686 (5.63%) |
| 4 |  | 45 of 1686 (2.67%) |
| 5 |  | 45 of 1686 (2.67%) |
| 6 |  | 43 of 1686 (2.55%) |
| 7 |  | 14 of 1686 (0.83%) |
| 8 |  | 3 of 1686 (0.18%) |
| 9 |  | 25 of 1686 (1.48%) |
| 10 |  | 1 of 1686 (0.06%) |

*Note*: ALG gap sizes = 40; AAG gap sizes = 30.

| 0 | 6318 of 9,825 (64.31%) | 476 of 1686 (28.23%) |
| --- | --- | --- |
| 1 | 2251 of 9,825 (22.91%) | 461 of 1686 (27.34%) |
| 2 | 801 of 9,825 (8.15%) | 292 of 1686 (17.32%) |
| 3 | 266 of 9,825 (2.71%) | 193 of 1686 (11.45%) |
| 4 | 170 of 9,825 (1.73%) | 60 of 1686 (3.56%) |
| 5 | 19 of 9,825 (0.19%) | 49 of 1686 (2.91%) |
| 6 |  | 15 of 1686 (0.89%) |
| 7 |  | 48 of 1686 (2.85%) |
| 8 |  | 23 of 1686 (1.36%) |
| 9 |  | 53 of 1686 (3.14%) |
| 10 |  | 15 of 1686 (0.89%) |
| 11 |  | 1 of 1686 (0.06%) |

*Note*: ALG gap sizes = 50; AAG gap sizes = 30.

| 0 | 5080 of 10,400 (48.85%) | 303 of 1686 (17.97%) |
| --- | --- | --- |
| 1 | 3014 of 10,400 (28.98%) | 370 of 1686 (21.95%) |
| 2 | 1486 of 10,400 (14.29%) | 431 of 1686 (25.56%) |
| 3 | 583 of 10,400 (5.61%) | 179 of 1686 (10.62%) |
| 4 | 113 of 10,400(1.09%) | 137 of 1686 (8.13%) |
| 5 | 55 of 10,400 (0.53%) | 68 of 1686 (4.03%) |
| 6 | 69 of 10,400 (0.66%) | 28 of 1686 (1.66%) |
| 7 |  | 33 of 1686 (1.96%) |
| 8 |  | 28 of 1686 (1.66%) |
| 9 |  | 51 of 1686 (3.02%) |
| 10 |  | 43 of 1686 (2.55%) |
| 11 |  | 9 of 1686 (0.53%) |
| 12 |  | 6 of 1686 (0.36%) |

*Note*: ALG gap sizes = 60; AAG gap sizes = 30.

| 0 | 3067 of 10,710 (28.64%) | 129 of 1686 (7.65%) |
| --- | --- | --- |
| 1 | 2942 of 10,710 (27.47%) | 179 of 1686 (10.62%) |
| 2 | 1783 of 10,710 (16.65%) | 333 of 1686 (19.75%) |
| 3 | 1384 of 10,710 (12.92%) | 216 of 1686 (12.81%) |
| 4 | 800 of 10,710 (7.47%) | 332 of 1686 (19.69%) |
| 5 | 513 of 10,710 (4.79%) | 128 of 1686 (7.59%) |
| 6 | 136 of 10,710 (1.27%) | 90 of 1686 (5.34%) |
| 7 | 14 of 10,710 (0.13%) | 43 of 1686 (2.55%) |
| 8 | 71 of 10,710 (0.66%) | 30 of 1686 (1.78%) |
| 9 |  | 42 of 1686 (2.49%) |
| 10 |  | 60 of 1686 (3.56%) |
| 11 |  | 41 of 1686 (2.43%) |
| 12 |  | 48 of 1686 (2.85%) |
| 13 |  | 14 of 1686 (0.83%) |
| 14 |  | 1 of 1686 (0.06%) |

*Note*: ALG gap sizes = 80; AAG gap sizes = 30.

| 0 | 1171 of 10,940 (10.70%) | 103 of 1686 (6.11%) |
| --- | --- | --- |
| 1 | 2958 of 10,940 (27.04%) | 108 of 1686 (6.41%) |
| 2 | 2269 of 10,940 (20.74%) | 225 of 1686 (13.35%) |
| 3 | 1096 of 10,940 (10.02%) | 228 of 1686 (13.52%) |
| 4 | 1188 of 10,940 (10.86%) | 212 of 1686 (12.57%) |
| 5 | 1038 of 10,940 (9.49%) | 198 of 1686 (11.74%) |
| 6 | 650 of 10,940 (5.94%) | 152 of 1686 (9.02%) |
| 7 | 314 of 10,940 (2.87%) | 108 of 1686 (6.41%) |
| 8 | 153 of 10,940 (1.40%) | 93 of 1686 (5.52%) |
| 9 | 103 of 10,940 (0.94%) | 39 of 1686 (2.31%) |
| 10 |  | 55 of 1686 (3.26%) |
| 11 |  | 26 of 1686 (1.54%) |
| 12 |  | 24 of 1686 (1.42%) |
| 13 |  | 14 of 1686 (0.83%) |
| 14 |  | 48 of 1686 (2.85%) |
| 15 |  | 47 of 1686 (2.79%) |
| 16 |  | 6 of 1686 (0.36%) |

*Note*: ALG gap sizes = 100; AAG gap sizes = 30.

| 0 | 715 of 11,213 (6.38%) | 48 of 1686 (2.85%) |
| --- | --- | --- |
| 1 | 1047 of 11,213 (9.34%) | 58 of 1686 (3.44%) |
| 2 | 1700 of 11,213 (15.16%) | 41 of 1686 (2.43%) |
| 3 | 684 of 11,213 (6.10%) | 47 of 1686 (2.79%) |
| 4 | 506 of 11,213 (4.51%) | 83 of 1686 (4.92%) |
| 5 | 647 of 11,213 (5.77%) | 205 of 1686 (12.16%) |
| 6 | 314 of 11,213 (2.80%) | 182 of 1686 (10.79%) |
| 7 | 329 of 11,213 (2.93%) | 136 of 1686 (8.07%) |
| 8 | 593 of 11,213 (5.29%) | 222 of 1686 (13.17%) |
| 9 | 689 of 11,213 (6.14%) | 128 of 1686 (7.59%) |
| 10 | 385 of 11,213 (3.43%) | 85 of 1686 (5.04%) |
| 11 | 307 of 11,213 (2.74%) | 95 of 1686 (5.63%) |
| 12 | 423 of 11,213 (3.77%) | 70 of 1686 (4.15%) |
| 13 | 409 of 11,213 (3.65%) | 36 of 1686 (2.14%) |
| 14 | 511 of 11,213 (4.56%) | 34 of 1686 (2.02%) |
| 15 | 446 of 11,213 (3.98%) | 62 of 1686 (3.68%) |
| 16 | 681 of 11,213 (6.07%) | 57 of 1686 (3.38%) |
| 17 | 389 of 11,213 (3.47%) | 37 of 1686 (2.19%) |
| 18 | 128 of 11,213 (1.14%) | 42 of 1686 (2.49%) |
| 19 | 279 of 11,213 (2.49%) | 13 of 1686 (0.77%) |
| 20 | 31 of 11,213 (0.28%) | 3 of 1686 (0.18%) |
| 21 |  | 1 of 1686 (0.06%) |
| 22 |  | 1 of 1686 (0.06%) |

*Note*: ALG gap sizes = 270; AAG gap sizes = 30.
