# Supplementary material for: Paleo-polyploidization in Lycophytes
Source: Genomics Proteomics Bioinformatics. 2020 Nov 4;18(3):333–40. doi: 10.1016/j.gpb.2020.10.002 (PMC7801247; doi:10.1016/j.gpb.2020.10.002)
Supplement: Supplementary Table S8 — Homology depth between S. lepidophylla and AAG genomes. [file mmc16.docx]

**Table S8 Homology depth between *S. lepidophylla* and AAG genomes**

| **Homologous depth level** | **AAG regions aligned to *S. lepidophylla*** | ***S. lepidophylla* regions aligned to AAG** |
| --- | --- | --- |
| 0 | 3247 of 14,249 (22.79%) | 87 of 1,590 (5.47%) |
| 1 | 2992 of 14,249 (21.00%) | 115 of 1,590 (7.23%) |
| 2 | 2745 of 14,249 (19.26%) | 115 of 1,590 (7.23%) |
| 3 | 2309 of 14,249 (16.20%) | 183 of 1,590 (11.51%) |
| 4 | 1515 of 14,249 (10.63%) | 199 of 1,590 (12.52%) |
| 5 | 1042 of 14,249 (7.31%) | 198 of 1,590 (12.45%) |
| 6 | 217 of 14,249 (1.52%) | 105 of 1,590 (6.60%) |
| 7 | 159 of 14,249 (1.12%) | 111 of 1,590 (6.98%) |
| 8 | 22 of 14,249 (0.15%) | 135 of 1,590 (8.49%) |
| 9 | 1 of 14,249 (0.01%) | 108 of 1,590 (6.79%) |
| 10 |  | 34 of 1,590 (2.14%) |
| 11 |  | 45 of 1,590 (2.83%) |
| 12 |  | 90 of 1,590 (5.66%) |
| 13 |  | 31 of 1,590 (1.95%) |
| 14 |  | 30 of 1,590 (1.89%) |
| 15 |  | 4 of 1,590 (0.25%) |

*Note*: *S. lepidophylla* gap sizes = 80; AAG gap sizes = 30.
